# Defective NOD2 peptidoglycan sensing promotes diet-induced inflammation, dysbiosis, and insulin resistance

**DOI:** 10.15252/emmm.201404169

**Published:** 2015-02-09

**Authors:** Emmanuel Denou, Karine Lolmède, Lucile Garidou, Celine Pomie, Chantal Chabo, Trevor C Lau, Morgan D Fullerton, Giulia Nigro, Alexia Zakaroff-Girard, Elodie Luche, Céline Garret, Matteo Serino, Jacques Amar, Michael Courtney, Joseph F Cavallari, Brandyn D Henriksbo, Nicole G Barra, Kevin P Foley, Joseph B McPhee, Brittany M Duggan, Hayley M O'Neill, Amanda J Lee, Philippe Sansonetti, Ali A Ashkar, Waliul I Khan, Michael G Surette, Anne Bouloumié, Gregory R Steinberg, Rémy Burcelin, Jonathan D Schertzer

**Affiliations:** 1 Department of Biochemistry and Biomedical Sciences, McMaster UniversityHamilton, ON, Canada; 2 Institut National de la Santé et de la Recherche Médicale (INSERM)Toulouse, France; 3 Université Paul Sabatier (UPS), Unité Mixte de Recherche (UMR) 1048, Institut des Maladies Métaboliques et Cardiovasculaires (I2MC), Team 1: «stroma-vascular cells of adipose tissue»Toulouse, France; 4 Université Paul Sabatier (UPS), Unité Mixte de Recherche (UMR) 1048, Institut des Maladies Métaboliques et Cardiovasculaires (I2MC), Team 2: «Intestinal Risk Factors, Diabetes, Dyslipidemia»Toulouse Cedex 4, France; 5 VAIOMER SAS, Prologue BiotechLabège, France; 6 Department of Medicine, McMaster UniversityHamilton, ON, Canada; 7 Unité de Pathogénie Microbienne Moléculaire and Unité INSERM 786, Institut PasteurParis, France; 8 Department of Pathology and Molecular Medicine, McMaster UniversityHamilton, ON, Canada; 9 Farncombe Family Digestive Health Research Institute, McMaster UniversityHamilton, ON, Canada

**Keywords:** diabetes, glucose, metabolic inflammation, microbiome, obesity

## Abstract

Pattern recognition receptors link metabolite and bacteria-derived inflammation to insulin resistance during obesity. We demonstrate that NOD2 detection of bacterial cell wall peptidoglycan (PGN) regulates metabolic inflammation and insulin sensitivity. An obesity-promoting high-fat diet (HFD) increased NOD2 in hepatocytes and adipocytes, and NOD2^−/−^ mice have increased adipose tissue and liver inflammation and exacerbated insulin resistance during a HFD. This effect is independent of altered adiposity or NOD2 in hematopoietic-derived immune cells. Instead, increased metabolic inflammation and insulin resistance in NOD2^−/−^ mice is associated with increased commensal bacterial translocation from the gut into adipose tissue and liver. An intact PGN-NOD2 sensing system regulated gut mucosal bacterial colonization and a metabolic tissue dysbiosis that is a potential trigger for increased metabolic inflammation and insulin resistance. Gut dysbiosis in HFD-fed NOD2^−/−^ mice is an independent and transmissible factor that contributes to metabolic inflammation and insulin resistance when transferred to WT, germ-free mice. These findings warrant scrutiny of bacterial component detection, dysbiosis, and protective immune responses in the links between inflammatory gut and metabolic diseases, including diabetes.

## Introduction

Obesity is associated with an elevation in the chronic inflammatory tone of metabolic tissues. This metabolic inflammation regulates glucose homeostasis, and the underlying host immune responses are emerging (Greiner & Bäckhed, 2011). Accumulation of immune cells such as neutrophils, lymphocytes, and macrophages in adipose and liver tissues during obesity are associated with augmented inflammatory mediators that contribute to insulin resistance (Weisberg *et al*, 2003; Elgazar-Carmon *et al*, 2008; Feuerer *et al*, 2009; Lumeng *et al*, 2009; Winer *et al*, 2009, 2011; Talukdar *et al*, 2012). The triggering mechanisms responsible for metabolic inflammation during obesity are ill-defined, but nutrient excess and bacterial origins have been proposed (Gregor & Hotamisligil, 2011; Nicholson *et al*, 2012). The ability of the gut microbiota from obese mice to predispose lean mice to increased weight gain has been linked to increased energy harvesting capacity (Turnbaugh *et al*, 2006; Bäckhed, 2012). However, the microbial origins of obesity-associated metabolic inflammation and integration of host immune and bacterial sensing strategies are only beginning to be appreciated (Bäckhed, 2011; Burcelin *et al*, 2012; Holmes *et al*, 2012). We previously showed that gut microbial imbalance (i.e., dysbiosis) controls a state of metabolic endotoxemia during obesity and that bacterial factors from the gut, such as lipopolysaccharide (LPS), accumulated in the blood and contributed to inflammation and insulin intolerance through CD14/TLR4 pathogen-sensing systems (Cani *et al*, 2007, 2008; Poggi *et al*, 2007; Luche *et al*, 2013).

Ablation of various pattern recognition receptors (PRRs) such as TLR4, CD14, PKR, and NLRP3 protect mice from diet-/obesity-induced inflammation and insulin resistance (Shi *et al*, 2006; Cani *et al*, 2007; Nakamura *et al*, 2010; Vandanmagsar *et al*, 2011). We previously showed that obese high-fat diet (HFD)-fed mice lacking both nucleotide oligomerization domain (NOD1) and NOD2 have reduced insulin resistance and inflammation (Schertzer *et al*, 2011). NOD1 and NOD2 are sensors of bacterial cell wall peptidoglycan (PGN) that elicit inflammation by augmenting cytokine, Paneth cell defensin (Stappenbeck *et al*, 2002), and stress kinase responses (Carneiro *et al*, 2008). We showed that PGN containing meso-DAP motifs (generally dominant in Gram-negative bacteria) caused profound insulin resistance through actions on NOD1 directly in metabolic cells, including adipocytes and hepatocytes (Schertzer *et al*, 2011). We also showed that NOD2 activation with the minimal bioactive PGN motif, muramyl dipeptide (MDP), more abundant in Gram-positive bacteria, elicited cell autonomous inflammation and impaired insulin action directly in muscle cells (Tamrakar *et al*, 2010) and could cause acute, transient, and peripheral insulin resistance *in vivo* (Schertzer *et al*, 2011). Hence, we originally hypothesized that lacking NOD2 detection of this type of PGN (i.e., MDP) would improve insulin sensitivity during obesity (Amar *et al*, 2011; Schertzer & Klip, 2011). We previously placed NOD2^−/−^ mice on a diabetogenic diet (containing 70% fat and less than 1% carbohydrate), which does not cause overt obesity. We did not consider that NOD2 deletion could worsen insulin resistance and the dietary stress during this 70% HFD was not suitable to find (genetic) factors that potentially worsen insulin action or glucose regulation. However, this former work did reinforce that deletion of NOD1, but not NOD2, provided some protection from glucose intolerance under these severe dietary conditions (Amar *et al*, 2011).

Metabolic differences due to deletion of NOD1 versus NOD2 would be consistent with the divergent results demonstrated by deletion of various NOD-like receptors in inflammasomes (Vandanmagsar *et al*, 2011; Henao-Mejia *et al*, 2012). Our current working model proposes that NOD1 deletion is protective (Amar *et al*, 2011), but to the best of our knowledge, no study has shown that defective PGN sensing by NOD2 may interact with a dietary stress to promote worse insulin resistance. This is a logical connection because defective NOD2 immunity has been associated with promoting other chronic pro-inflammatory pathologies, dysbiosis and human NOD2 variants have the highest risk association with Crohn's disease (Ogura *et al*, 2001). NOD2 immunity may also to contribute to gut microbial homeostasis, but the relevance to metabolic disease and insulin resistance has not yet been fully explored (Rehman *et al*, 2011). We demonstrate here that defective NOD2 sensing of PGN, either by NOD2 gene deletion or by mutations in PGN, promotes an increased bacterial invasion of metabolic tissues associated with inflammation and insulin resistance. Furthermore, the dysbiosis in mice continually bred with a NOD2-deletion plus the stress of an obesity-causing diet is an independent and transmissible factor that contributes to increased metabolic inflammation and insulin resistance.

## Results

### NOD2 deletion exacerbates HFD-induced insulin resistance

**Figure 1:**
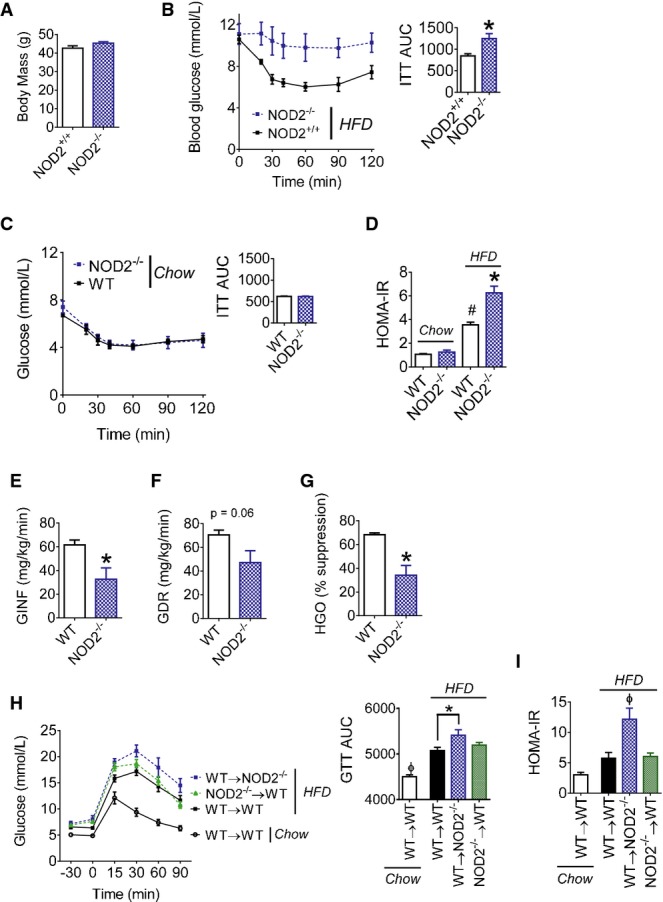
NOD2 deletion in mice exacerbates diet-induced insulin resistance
A, B Body mass (A), blood glucose, and the cumulative area under the curve (AUC) (B) during insulin tolerance tests (ITT; 1.0 IU/kg i.p.) in NOD2^+/+^ (*n *=* *8) and NOD2^−/−^ (*n *=* *10) littermate mice fed a HFD for 16 weeks, *P =* *0.001.C Blood glucose and the cumulative AUC during insulin tolerance tests (ITT; 0.5 IU/kg i.p.) in chow-fed WT (*n *=* *6) and NOD2^−/−^ (*n *=* *6) mice.D HOMA insulin resistance (IR) index in weight-matched chow- (*n *=* *7) or HFD-fed (*n *=* *10) WT and NOD2^−/−^ mice, ^#^*P *=* *0.0001 (WT chow versus WT HFD) and *P =* *0.0001 (WT HFD versus NOD2^−/−^ HFD).E, F Glucose infusion rate (GINF) (E) and glucose disposal rate (GDR) (F) during hyperinsulinemic euglycemic clamps in weight-matched WT (*n *=* *4) and NOD2^−/−^ (*n *=* *3) mice fed a HFD for 16 weeks, *P =* *0.02.G Percentage of hepatic glucose output (HGO) suppression during hyperinsulinemic euglycemic clamps in weight-matched WT (*n *=* *4) and NOD2^−/−^ (*n *=* *3) mice fed a HFD for 16 weeks, *P =* *0.005.H Blood glucose and cumulative AUC during glucose tolerance tests (GTT; 1.0 g/kg) in chow-fed (*n *=* *3) or HFD-fed WT and NOD2^−/−^ mice (*n *>* *8 for all groups) after bone marrow transplantation, *P = 0.04 and ^ϕ^*P* = 0.01.I HOMA-IR in chow-fed (*n *=* *3) or HFD-fed WT and NOD2^−/−^ mice (*n *>* *8 for all groups) after bone marrow transplantation, ^ϕ^*P* = 0.003.
Data information: *Significantly different from HFD-fed NOD2^+/+^ or WT mice or as indicated. ^#^Significantly different from WT chow-fed mice. ^ϕ^Significantly different from all other conditions. An unpaired *t*-test was used for comparisons between two conditions, whereas a 1-way ANOVA was used for comparisons between more than two conditions. Tukey's post-hoc test was used. Values are mean ± SEM.

First-generation NOD2^+/+^ and NOD2^−/−^ littermates (bred from in-house NOD2-heterozygote parents) showed that deletion of NOD2 exacerbated insulin intolerance, independent of any difference in body mass, when the mice were fed a HFD for 16 weeks (Fig1A and B). We used non-littermate mice for the remainder of the experiments. In order to specifically investigate diet-induced insulin resistance, we weight-matched WT and NOD2^−/−^ mice after 16 weeks on the HFD. Overall, we did not find any metabolic differences in chow-fed wild-type (WT) and NOD2^−/−^ mice, which had similar insulin tolerance and HOMA-IR (Fig1C and D). However, HFD-fed NOD2^−/−^ mice had exacerbated glucose tolerance (Supplementary Fig S1A–C) and higher HOMA-IR compared to weight-matched WT mice (Fig1D). Hyperinsulinemic euglycemic clamps showed that weight-matched HFD-fed NOD2^−/−^ mice had exacerbated whole body, hepatic insulin resistance determined by lower rates of glucose infusion (GINF), and lower insulin-induced suppression of hepatic glucose output (HGO), respectively (Fig1E–G; Supplementary Fig S1D–F). We next generated mice that lacked NOD2 in the hematopoietic or non-hematopoietic compartments using lethal irradiation and bone marrow reconstitution. HFD-fed NOD2^−/−^ mice that were reconstituted with WT bone marrow had worse glucose tolerance during a GTT and a higher HOMA-IR index when compared to WT mice that were reconstituted with either WT or NOD2-deficient bone marrow (Fig1H and I). Therefore, NOD2 deletion in non-hematopoietic cells equated to worse glucose control during a HFD.

### NOD2 deletion exacerbates diet-induced adipose tissue inflammation

**Figure 2:**
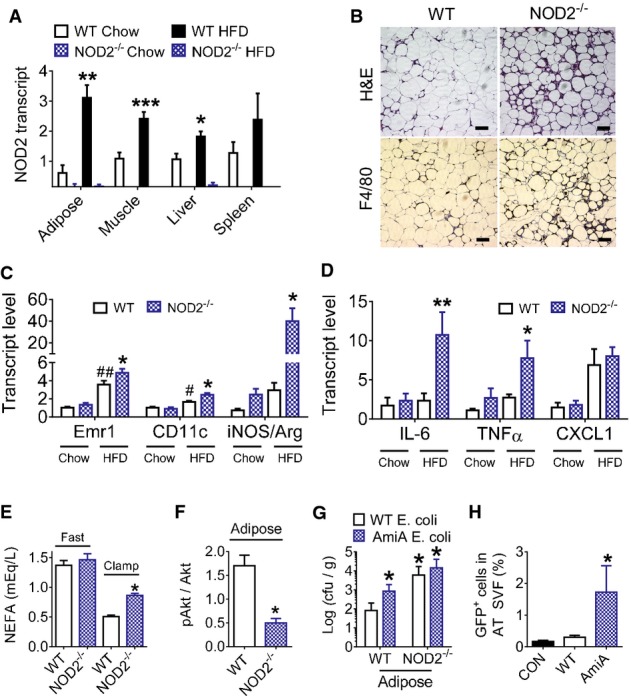
Defects in NOD2 sensing of PGN promote gut bacterial translocation to adipose tissue and diet-induced adipose inflammation and dysfunction
A Transcript levels of NOD2 in various tissues of chow-fed (*n *=* *5, all tissues in both genotypes) and 16 week HFD-fed (*n *=* *6) WT and NOD2^−/−^ mice, *P =* *0.005, **P =* *0.001, and ***P =* *0.0004.B H&E histology and IHC of the macrophage marker F4/80 in adipose tissue from HFD-fed WT and NOD2^−/−^ mice (representative of *n *=* *5 WT and *n *=* *6 NOD2^−/−^ mice). Scale bar equals 50 μm.C, D Quantification of immune cell and inflammatory markers (C) and pro-inflammatory cytokines (D) in adipose tissue of chow-fed (*n *=* *5 in both genotypes) or HFD-fed (*n *=* *11 in both genotypes) WT and NOD2^−/−^ mice, *P =* *0.04, **P =* *0.02, ^##^*P *=* *0.004, and ^#^*P *=* *0.04.E Quantification of serum non-esterified fatty acids (NEFA) in fasted and clamped HFD-fed WT (*n *=* *4) and NOD2^−/−^ (*n *=* *3) mice, *P =* *0.0001.F Quantification of insulin-stimulated pAkt^Ser473^ in gonadal adipose tissue at the end of the clamp in HFD-fed WT (*n *=* *5) and NOD2^−/−^ mice (*n *=* *5), *P = 0.04.G The number of ampicillin-resistant *E. coli* cfu per gram of visceral adipose tissue was determined in WT (*n *=* *5) and NOD2^−/−^ (*n *=* *5) mice after oral administration of 10^9^ cfu of WT or mutant (*ΔamiA*) DsRed-labeled *E. coli*, *P =* *0.02.H Quantification of GFP-positive *E. coli* in the adipose tissue stromal vascular fraction (SVF) from WT mice with no oral bacterial administration (CON,*n *=* *3) or after administration of 10^9^ cfu WT GFP-positive *E. coli* (*n *=* *4) or *ΔamiA*GFP-positive *E. coli* (*n *=* *4), *P =* *0.03.
Data information: In (A), *significantly different from chow-fed WT mice. In (C–F), *significantly different from HFD-fed WT mice. In (G), *significantly different from WT mice given WT *E. coli*. In (H), *significantly different from CON plus WT *E. coli* given to WT mice. ^#^Significantly different from chow-fed WT mice. An unpaired *t*-test was used for comparisons between two conditions, whereas a 1-way ANOVA was used for comparisons between more than two conditions. Tukey's post-hoc test was used. Values are mean ± SEM.

We found that a HFD increased NOD2 transcript levels in adipose tissue, muscle, and liver of WT mice and confirmed that NOD2^−/−^ mice did not express NOD2 mRNA (Fig2A). NOD1 transcript levels were not altered by genotype or diet in these tissues, and there were some diet-induced changes in other PRRs seen in the adipose and liver tissues (Supplementary Fig S1G–J). Within adipose tissue, the HFD increased NOD2 transcript levels in adipocytes (Supplementary Fig S2A). An abundance of non-adipocyte cells (dark stained by H&E) and macrophages (F4/80^+^) were detectable in the adipose tissue in HFD-fed NOD2^−/−^ mice (Fig2B; Supplementary Fig S2B). Higher transcript levels of adipose tissue Emr1 and CD11c were detected after the HFD, which were both significantly augmented in HFD-fed NOD2^−/−^ mice compared to HFD-fed WT mice (Fig2C). Transcript levels of IL-6 and TNF-α were elevated, and the ratio of iNOS/Arginase was elevated by more than 30-fold in adipose tissue of HFD-fed NOD2^−/−^ mice (Fig2C and D). Chow-fed WT and NOD2^−/−^ mice were not different for these inflammatory markers (Fig2C and D). Flow cytometry showed that NOD2^−/−^ mice had higher macrophage and dendritic-like cells and higher CD3^+^ lymphocytes in the stromal vascular fraction (SVF) after 4 weeks on a very high-fat diet containing 70% Kcal from fat (Supplementary Fig S2C–F). Changes in inflammation were associated with defective adipose tissue function. For example, the ability of insulin to suppress lipolysis was blunted in HFD-fed NOD2^−/−^ mice, since serum NEFA was higher during the clamp (Fig2E). Adipose tissue also had defective insulin-induced signaling at the level of Akt phosphorylation (Fig2F; Supplementary Fig S2K).

### Defective NOD2 sensing of PGN promotes bacterial infiltration into metabolic tissues

In order to define a potential trigger for excessive inflammation, we next determined if defective host NOD2 sensing of bacterial PGN precipitated microbial translocation into adipose tissue. To determine the importance of PGN sensing, without the potentially confounding variable of a HFD, we orally administered WT or PGN *ΔamiA* mutant mouse *Escherichia coli* expressing GFP (and DSRed) and beta lactamase (to provide ampicillin resistance) in chow-fed WT and NOD2^−/−^ mice. Alteration of *amiA* gene in *E. coli* suppresses the production of cell division amidases and alters the PGN exoskeleton resulting in decreased muropeptide turnover and weaker NOD2-mediated immune responses (Yang *et al*, 2012). Two hours after gavage, visceral adipose tissue from all mice given *ΔamiA E. coli* had significantly higher number of ampicillin-resistant colonies compared to after gavage with WT *E. coli* (Fig2G). Similarly, adipose tissue from NOD2^−/−^ mice had increased the abundance of ampicillin-resistant colonies compared to WT mice, when all mice were gavaged with WT *E. coli* (Fig2G). Similar results were obtained if bacterial DNA or RNA were measured in adipose tissue (Supplementary Fig S2I and J). These data show that both bacterial PGN structure and host NOD2-immunity regulate penetration of a commensal mouse bacterium into adipose tissue. Importantly, the measurements of DNA, RNA, or colony-forming units (cfu) were designed to detect the orally delivered *E. coli* and all of these measurements were undetectable in the adipose tissue of non-gavaged mice. After oral delivery of *E. coli,* WT mice also showed a significant increase in adipose-tissue-resident stromal vascular fraction (SVF) cells containing GFP-positive *ΔamiA E. coli* compared to GFP-positive WT *E. coli* (Fig2H; Supplementary Fig S2G and H). Hence, NOD2 sensing of PGN protected against bacterial translocation in adipose tissue, where adipose SVF cells promote or respond to adipose tissue microbiota.

**Figure 3:**
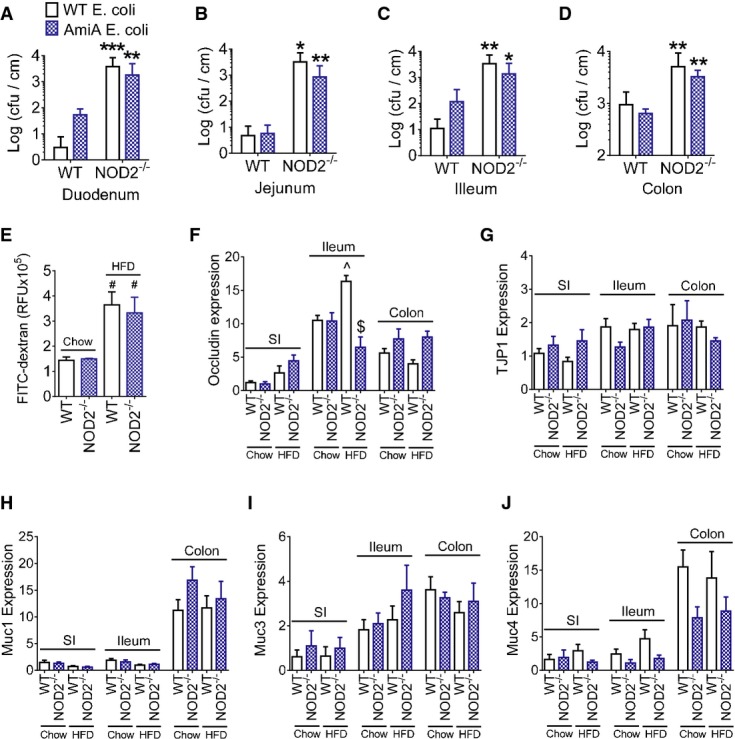
Defects in NOD2 sensing of PGN promote excessive intestinal bacterial adherence
A-D The number of ampicillin-resistant *E. coli* cfu per cm of gut mucosa in the duodenum (A), jejunum (B), ileum (C), and colon (D) 2 h after oral administration of 10^9^ cfu of WT *E. coli* in WT (*n *=* *4) and NOD2^−/−^ (*n *=* *6) mice or *ΔamiA E. coli* in WT (*n *=* *5) and NOD2^−/−^ (*n *=* *6) mice. *Significantly different from WT mice, *P =* *0.04, **P =* *0.02, and ***P =* *0.01.E FITC detected in the serum 4 h after oral gavage of FITC-dextran in chow-fed WT (*n *=* *4) and NOD2^−/−^ mice (*n *=* *3) and HFD-fed WT (*n *=* *4) and NOD2^−/−^ mice (*n *=* *5) mice, ^#^Significantly different from chow-fed mice, *P *=* *0.02.F-J Quantification of tight junction markers occludin (F) and TJP1 (G) and mucins (H–J) in the small intestine (SI), illeum, and colon of chow-fed WT (*n *=* *9), chow-fed NOD2^−/−^ (*n *=* *8), HFD-fed WT (*n *=* *9), and HFD-fed NOD2^−/−^ mice (*n *=* *7). ^^^Significantly different from chow-fed mice in the same gut segment, *P *=* *0.002. ^$^Significantly different from WT HFD-fed mice in the same gut segment, *P *=* *0.0001.
Data information: A 1-way ANOVA was used for comparisons between more than two conditions. Tukey's post-hoc test was used. Values are mean ± SEM.

In order to implicate NOD2 actions in the gut that could precipitate bacterial accumulation in adipose tissue, we next determined if host NOD2 sensing of bacterial PGN alters bacterial colonization and adherence in the intestine. Two hours after gavage with either WT *E. coli* or *ΔamiA E. coli*, bacterial accumulation was higher in all of the gut segments examined in NOD2^−/−^ mice compared to WT mice (Fig3A–D). Gut permeability to FITC-dextran was not altered in NOD2^−/−^ mice, but was increased in all HFD-fed mice (Fig3E). Expression of the tight junction component, occludin, was increased in the ileum of HFD-fed WT mice, but in comparison, HFD-fed NOD2^−/−^ mice had lower occludin transcript levels (Fig3F). Neither diet nor NOD2 genotype altered the expression of tight junction protein 1 (TJP1) or selected mucins in various gut segments of mice (Fig3G–J), including no difference in Muc2 levels in HFD-fed WT or NOD2^−/−^ mice (Supplementary Fig S2L).

**Figure 4:**
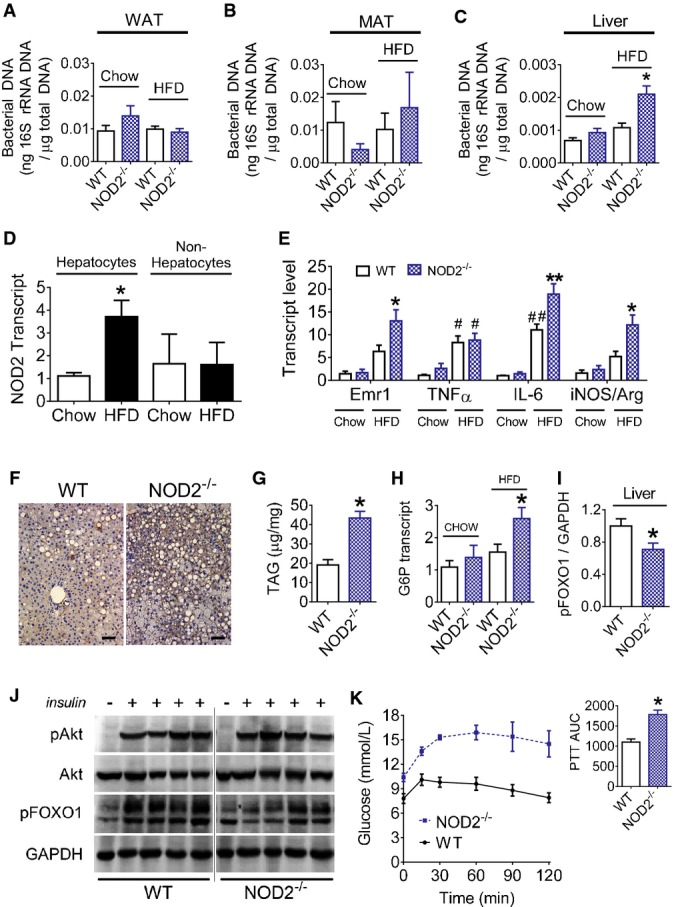
NOD2 deletion exacerbates diet-induced bacterial translocation, inflammation, and impairs insulin action in the liver
A-C Quantification of bacterial DNA in the gonadal white adipose tissue (A) (WAT,*n *=* *14–16 mice for all groups), mesenteric adipose tissue (B) (MAT,*n *=* *10–15 mice for all groups), and liver (C) (*n *=* *10–12 mice for all groups) of chow-fed and 16 week HFD-fed WT and NOD2^−/−^ mice, *P =* *0.0002.D Quantification of NOD2 transcripts in hepatocyte and non-hepatocyte cell populations of liver from chow-fed (*n *=* *10) and HFD-fed (*n *=* *6) WT mice, *P =* *0.006.E Quantification of macrophage and inflammatory markers in liver of chow-fed (*n *=* *5 in both genotypes) or HFD-fed (*n *=* *11 in both genotypes) WT and NOD2^−/−^ mice (*n *=* *6 for IL-6 analysis), *P =* *0.02, **P =* *0.0001, ^#^*P *=* *0.02, and ^##^*P *=* *0.002.F Representative liver IHC for the macrophage marker F4/80 (representative of *n *=* *5 WT and *n *=* *6 NOD2^−/−^ mice). Scale bar equals 50 μm.G Quantification of hepatic triglycerides (TAG) in HFD-fed WT (*n *=* *6) and NOD2^−/−^ (*n *=* *6) mice, *P =* *0.0002.H Quantification of hepatic G6P transcript levels in chow-fed (*n *=* *5) and HFD-fed (*n *=* *11) WT and NOD2^−/−^ mice, *P =* *0.02.I, J Quantification (I) and immunoblots (J) of insulin-stimulated pFOXO1^Ser256^ in liver lysates after vena cava injection of insulin (0.5 IU/kg) in HFD-fed WT (*n *=* *4) and NOD2^−/−^ (*n *=* *4) mice, *P =* *0.0498.K Blood glucose and quantification of the AUC during a 120-min pyruvate tolerance test (PTT, 2.0 g/kg pyruvate, i.p.) in HFD-fed WT (*n *=* *6) and NOD2^−/−^ mice (*n *=* *6), *P =* *0.0005.
Data information: An unpaired *t*-test was used for comparisons with two conditions, whereas a 1-way ANOVA was used for comparisons with more than two conditions. Tukey's post-hoc test was used. *Significantly different from HFD-fed WT mice; In (D), *significantly different from chow-fed, WT mice. ^#^Significantly different from chow-fed WT mice. Values are mean ± SEM.

Given these proof-of-principle experiments in adipose tissue and that deletion of NOD2 favors an increase in the opportunity for adherence of orally delivered bacteria to intestinal mucosa, we next examined whether this promoted bacterial translocation during the same HFD that elicited augmented inflammation and insulin resistance in NOD2^−/−^ mice. We found that HFD-fed NOD2^−/−^ mice have increased bacterial DNA in the liver, but not adipose tissue depots (Fig4A–C). A HFD increased NOD2 transcript levels in hepatocytes, but not non-hepatocyte cells in the livers of WT mice (Fig4D). The increased bacterial load in the liver also coincided with augmented transcript levels of Emr1, IL-6, and increased iNOS/Arginase ratio in the livers of HFD-fed NOD2^−/−^ mice (Fig4E). HFD-fed NOD2^−/−^ mice had increased liver-resident (F4/80^+^) macrophages and increased steatosis evident by histology (Fig4F) and higher TAGs (Fig4G). Consistent with increased HGO during the clamp, HFD-fed NOD2^−/−^ mice had higher transcript levels of glucose 6-phosphatase (G6P), lower hepatic insulin-stimulated signaling at the level of FOXO1 phosphorylation, and higher blood glucose after a pyruvate challenge (Fig4H–K).

### Gut microbiota contributes to poor glucose control in the absence of NOD2

**Figure 5:**
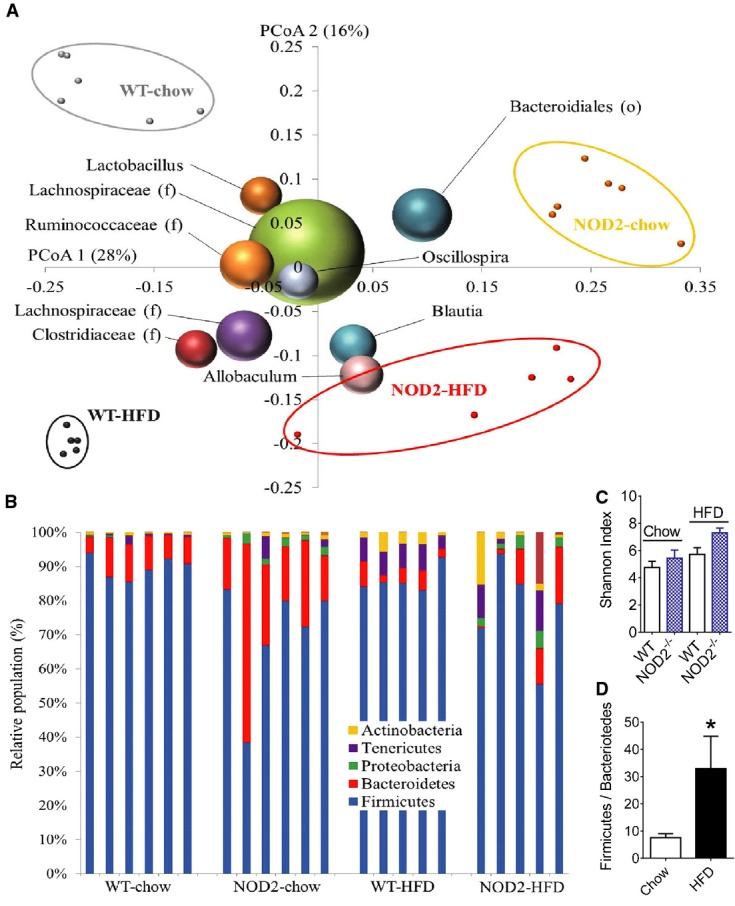
Diet and NOD2 influence the gut microbiome
A Unweighted UniFrac principal coordinates analysis (PCoA) plot illustrating the relative degree that genotype (NOD2^−/−^ versus WT, PCoA1: 28%) and diet (chow versus HFD, PCoA2: 16%) defined the diversity of the microbiota in the cecum. The most abundant bacterial genus are labeled and superimposed on the same PCoA plot. Each small dot is a mouse, and the size of the sphere representing a taxon is proportional to the mean relative abundance of the taxon across all samples.B Bar graph showing the relative abundance of phyla from each mouse cecum, where each bar is a separate mouse.C Alpha diversity represented by the Shannon index in chow-fed (*n *=* *6) and HFD-fed (*n *=* *5) WT and NOD2^−/−^ mice. Values are mean ± SEM.D Impact of diet on the Firmicutes/Bacteroidetes ratio in the cecum of chow-fed (*n *=* *12) and HFD-fed (*n *=* *10) mice. *Significantly different from chow-fed mice, *P *=* *0.03. An unpaired *t*-test was used. Values are mean ± SEM.

Given our findings on bacterial intestinal adherence and invasion into metabolic tissues, we next determined if intestinal dysbiosis contributed to glucose homeostasis in HFD-fed NOD2^−/−^ mice. We first showed that a dietary stress (HFD) and genotype (NOD2) influenced the microbial composition in the cecum at the phylum and genus levels (Fig5A and B). Principal coordinate analysis (PCoA) of unweighted UniFrac distances (Lozupone *et al*, 2011) based on operational taxonomic units (OTUs) confirmed that mice clustered differently based on their genotype and their diet (Fig5A). Cluster analysis demonstrated that the first coordinate (28%) distinguished mice based on the genetics of the host, whilst the second coordinate (16%) differentiated based on the diet (Fig5A). There were no differences in the alpha diversity (Shannon diversity index) related to diet and/or genotype, but we did demonstrate the expected increase in the Firmicutes to Bacteriodetes ratio associated with the HFD (Fig5B–D). The microbial composition of the cecum was not strikingly different at phylum and class level, but some differences were found at family and genus level within groups. Genotype alone defined the prevalence of Rikinellaceae, *Alistipes, Desulfovibrio, Bilophila,* and *Dehalobacterium,* whilst HFD diet increased both *Blautia* and *Streptococcus* compared to chow-fed mice (Supplementary Fig S3A and B). In particular, only HFD-fed NOD2^−/−^ mice displayed higher abundance of *Helicobacter* and Peptococcaceae when compared to all other mice and a lower prevalence of *Clostridium* when compared to HFD-fed WT mice (Supplementary Fig S3C).

**Figure 6:**
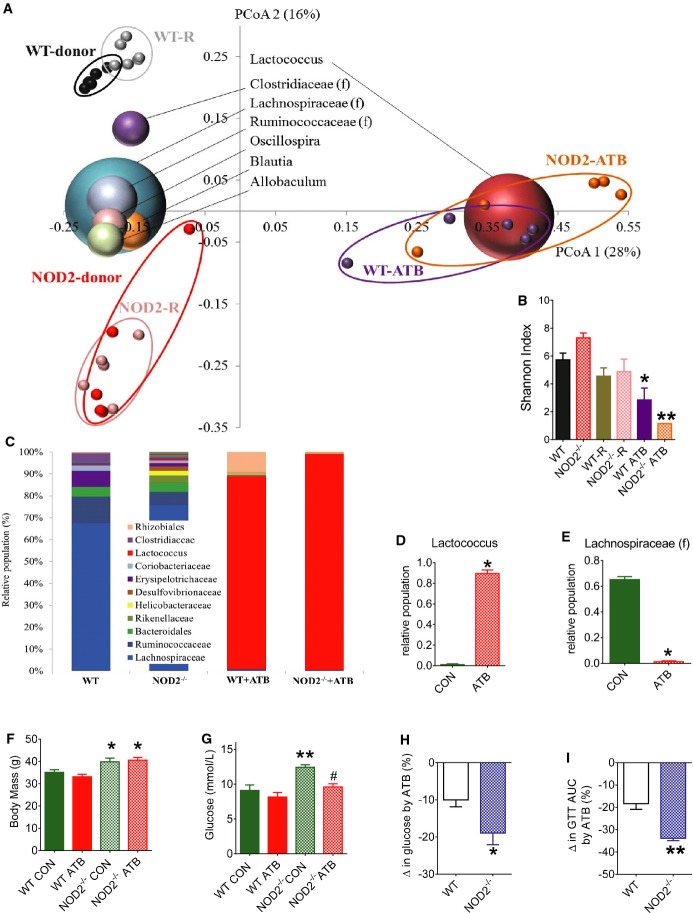
Antibiotics remove NOD2^−/−^-associated dysbiosis contributing to insulin resistance
A Principal coordinates analysis (PCoA) performed on pairwise unweighted UniFrac distances in HFD-fed mice shows a strong antibiotic effect (PCoA1: 28%) and a strong similarity within donors and recipients (R) from the microbiota transfer experiment into germ-free mice (PCoA2: 16%). All mice were fed a HFD, each small dot is a mouse, and the most abundant bacterial genus are labeled and superimposed on the same PCoA plot. The size of the sphere representing a taxon is proportional to the mean relative abundance of the taxon across all samples.B Alpha diversity represented by the Shannon index (*n ≥ *5/group), *P =* *0.03 and **P =* *0.0001.C Average relative abundance of the major genus in the cecum of each group of mice (*n ≥ *5/group).D, E Impact of antibiotics (ATB) on relative population of *Lactococcus* genus and Lachnospiraceae family in all mice (E) (*n ≥ *10/group), *P = 0.0001.F, G Body mass (F) and fasting blood glucose (G) in 16 week HFD-fed WT and NOD2^−/−^ mice without (CON) or with ATB in the drinking water for the final 4 weeks of the HFD (*n ≥ *5/group), *P =* *0.04, **P =* *0.0008, and ^#^*P *=* *0.004.H, I Percentage change in fasting blood glucose (H) and AUC during GTTs (I) that was induced by ATB in HFD-fed WT and NOD2^−/−^ mice (*n ≥ *5/group), *P =* *0.04 and **P =* *0.005.
Data information: *Significantly different from HFD-fed WT mice: ^#^Significantly different from HFD-fed NOD2^−/−^ CON mice (without ATB). An unpaired *t*-test was used for comparisons with two conditions, whereas a 1-way ANOVA was used for comparisons with more than two conditions. Tukey's post-hoc test was used. Values are mean ± SEM.

We next attempted to eliminate these microbial differences by giving antibiotics to HFD-fed mice. Principal coordinate analysis of unweighted UniFrac distances showed primary clustering (PCoA1 (28%)) of cecal microbiota from all mice given antibiotics (1 g/ml ampicillin and 0.5 g/ml neomycin) in the drinking water for 4 weeks (Fig6A). Without antibiotics, HFD-fed WT and NOD2^−/−^ mice diverged into two distinct clusters through the second coordinate (PCoA 2 (16%)) (Fig6A). The microbial α-diversity determined by the Shannon index was lower in both groups of HFD-fed mice given antibiotics (Fig6B). Antibiotic treatment resulted in Lactococcus genus becoming the dominant group instead of Lachnospiraceae family, and essentially removed differences in the cecal microbiome of HFD-fed WT and NOD2^−/−^ mice, albeit a completely different microbial community during the provision of antibiotics (Fig6C–E; Supplementary Fig S4A). Importantly, antibiotic treatment did not alter body mass (Fig6F) or adipose tissue mass (Supplementary Fig S4E). However, antibiotic treatment reduced fasting blood glucose only in HFD-fed NOD2^−/−^ mice, but not WT mice (Fig6G and H). Antibiotic treatment lowered the AUC during a GTT (i.e., improved glucose tolerance) to a greater extent in HFD-fed NOD2^−/−^ mice compared to antibiotic treatment in HFD-fed WT mice (Fig6I; Supplementary Fig S4B–D).

**Figure 7:**
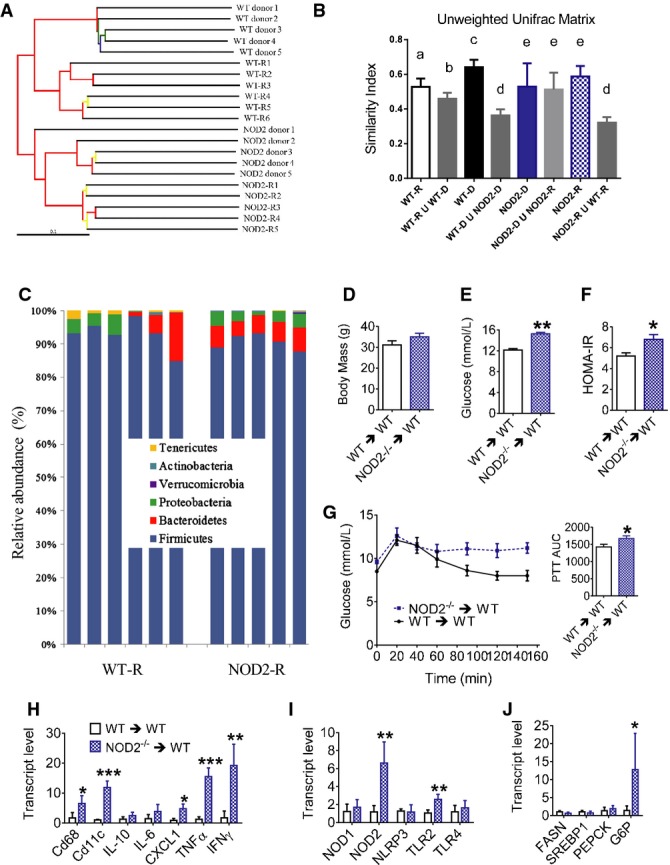
Transfer of HFD-fed NOD2^−/−^ microbiota contributes to inflammation and insulin resistance
A Hierarchical cluster tree built using UPGMA (unweighted pair group method with arithmetic mean) based on the unweighted UniFrac distance matrix. Each branch represents a numbered mouse from each group of WT donor, WT-R (WT recipient, which are WT germ-free mice that received WT donor flora), NOD2^−/−^ donor, or NOD2-R (NOD2 recipient, which are WT germ-free mice that received NOD2^−/−^ donor flora). Branches in the UPGMA tree are colored according to their jackknife support: red, 75–100%; yellow, 50–75%; green, 25–50%; and blue, < 25% support.B Calculation of the unweighted UniFrac matrix similarity index showed the level of successful transfer of the microbiome to recipient mice evidenced by results comparing the similarity within each group to the union (U) between different conditions. Conditions with different letters (a, b c, d, e) in the similarity index denote a statistical difference compared to all other conditions without the same letter, where the minimal statistical values for all comparisons are: ^a^*P* = 0.01, ^b^*P* = 0.04, ^c^*P* = 0.004, ^d^*P* = 0.0001, and ^e^*P* = 0.002.C Relative abundance of phyla in the cecum from each recipient mouse.D Body mass in weight-matched HFD-fed WT-R (*n *=* *5) and NOD2-R (*n *=* *4) mice, 5 weeks after colonization of germ-free mice.E, F Fasting blood glucose (E) and HOMA insulin resistance (IR) index (F) in weight-matched HFD-fed WT-R (*n *=* *5) and NOD2-R (*n *=* *4) mice, 5 weeks after colonization of germ-free mice, *P =* *0.02 and **P =* *0.0001.G Blood glucose and quantification of the AUC during a PTT in HFD-fed WT-R (*n *=* *7) and NOD2-R (*n *=* *6) mice, *P =* *0.01.H, I Quantification of immune cell and inflammatory markers (H) and PRRs (I) in adipose tissue of weight-matched HFD-fed WT-R (*n *=* *5) and NOD2-R (*n *=* *4) mice, *P =* *0.01, **P =* *0.001, and ***P =* *0.0001.J Quantification of metabolic transcripts in the liver of weight-matched HFD-fed WT-R (*n *=* *5) and NOD2-R (*n *=* *4) mice, *P =* *0.04.
Data information: *Significantly different from mice colonized with WT microbiota. An unpaired *t*-test was used for comparisons between two conditions, whereas a 1-way ANOVA was used for comparisons between more than two conditions. Tukey's post-hoc test was used. Values are mean ± SEM.

We next determined if the microbiota regulated inflammation and metabolism independently of host genetics. Hence, we evaluated the transmissibility of the NOD2-related insulin resistance phenotype to WT germ-free mice, where all donor and recipient mice were fed a HFD (Supplementary Fig S5A). The donor mice used in this experiment were the same HFD-fed WT and NOD2^−/−^ mice from Fig5, which allowed us to directly compare the microbiome of all conditions (Figs6A and 7A and B). PCoA using unweighted UniFrac distances showed a distinct clustering of the WT versus NOD2^−/−^ donor and respective recipient, WT germ-free mice, primarily through the second coordinates (PCoA 2 (16%)) (Fig6A). For example, the WT germ-free mice that were reconstituted with the microbiota from NOD2^−/−^ HFD-fed mice (i.e., NOD2-R) grouped closely with these NOD2-donors. The microbial α-diversity (Shannon diversity index) was similar between the donor and recipient flora (Fig6B). Segregation of WT donor and recipient microbial profiles from those in NOD2^−/−^ donors, and recipients were also evident when a jackknifed hierarchical cluster tree was generated using UPGMA calculation based on the unweighted UniFrac distance, which produced the same patterns inferred from the PCoA plots (Fig7A). Firmicutes and Bacteroidetes were the major phyla all recipient mice (Fig7C). Transfer of specific bacterial genus from donors to respective recipient mice was evident and highlighted the absence of cross-contamination within the different groups of recipient mice (>Supplementary Fig S5D–F). These transferred genus specific to NOD2^−/−^ microbiota included *Alistipes,* Rikenellaceae, *Desulfovibrionaceae* (*f*), *Desulfovibrio* and *Bacteroides*, and Clostridium related to WT microbiota (Supplementary Fig S5D). Transfer efficiency of the microbiota to WT germ-free mice was quantified using the similarity index based on unweighted UniFrac distances, and NOD2^−/−^ donor mice and recipient WT mice shared the same distances within each group and the union (U) between the groups demonstrating similarity in these microbial profiles (Fig7B). Transfer from the WT donor and WT recipient mice was also successful since the distance between WT donors and WT recipients was less than between WT donor and NOD2 donor as well as between WT recipient and NOD2 recipient (Fig7B). Overall these data show that the transfer of microbial community was successful and specific from donors of either genotype to germ-free WT recipients. Notably, body weight-matched mice that received the NOD2^−/−^ HFD-fed microbiota had higher fasting blood glucose and higher insulin resistance measured by the HOMA insulin resistance (IR) index (Fig7D–F). Also, mice that received the NOD2^−/−^ HFD-fed microbiota had higher glucose levels during a pyruvate challenge (Fig7G), indicative of higher gluconeogenesis. Adipose tissue from WT mice that received the NOD2^−/−^ microbiota had increased transcript levels of markers of immune cell infiltration (Cd68, Cd11c) and certain inflammatory cytokines (CXCL1, TNF-α, IFN-γ) (Fig7H), higher NOD2 transcript levels (Fig7I), and a striking increase in hepatic G6P transcript levels (Fig7J). Muscle and liver did not show changes in markers of immune cell infiltration, inflammation and bacterial translocation into metabolic tissues was not different after microbiota transfer (Supplementary Fig S5B and C). Thus, transfer of the microbiota alone from HFD-fed NOD2^−/−^ mice recapitulates only part of the inflammation and glucose homeostasis effects seen in HFD-fed NOD2^−/−^ mice.

## Discussion

Obesity skews metabolic tissues governing glucose homeostasis toward a pro-inflammatory status, which involves inputs from nutritional factors and the gut microbiota (Bäckhed *et al*, 2007; Cani *et al*, 2007, 2008; Velagapudi *et al*, 2010; Greiner & Bäckhed, 2011; Nicholson *et al*, 2012; Tremaroli & Backhed, 2012; Fullerton *et al*, 2013). PRRs appear to be a point of convergence in sensing metabolic and bacterial sources of inflammation during obesity. TLRs and NOD-like receptors (NLRs) can contribute to augmented inflammatory tone and impaired insulin action in response to classical bacteria-derived pathogen motifs and lipid metabolites (Senn, 2006; Shi *et al*, 2006; Schertzer *et al*, 2011; Vandanmagsar *et al*, 2011; Wen *et al*, 2011).

We here provide molecular evidence regarding the role of bacterial cell wall detection in the potential triggering mechanisms linking gut bacteria and metabolic inflammation since we demonstrate that: (1) absence of NOD2 in non-hematopoietic cells exacerbates insulin resistance during a HFD, (2) a NOD2-dependent peptidoglycan sensing system is necessary to limit accumulation of bacteria and inflammation in metabolic tissues, and (3) gut microbiota dysbiosis during HFD-feeding in the absence of NOD2 is an independent and transmissible factor contributing to metabolic inflammation and insulin resistance. Our results add NOD2 as an important PRR to the growing list of immune components that protect against dysbiosis and metabolic disease during obesity, which includes TLR2 (Caricilli *et al*, 2011), TLR5 (Vijay-Kumar *et al*, 2010), and inflammasome components (Henao-Mejia *et al*, 2012).

NOD2^−/−^ mice have impaired gut barrier function (Barreau *et al*, 2010). A HFD increased gut permeability to FITC-dextran, but we found no overt defect in gut permeability (to FITC-dextran) in NOD2^−/−^ mice. However, using host NOD2 deletion or by modifying the structure of bacterial PGN, we show that a defective NOD2/PGN sensing system fosters increased opportunity for bacterial adherence/colonization of the intestine. We show here that a defective PGN/NOD2 sensing system increased translocation of live (GFP-positive) bacteria into the SVF of adipose tissue. After these proof-of-principle experiments, we found that a 16-week HFD commonly used to cause insulin resistance increased bacterial DNA in the liver of only NOD2^−/−^ mice. This demonstrates that NOD2 deletion promotes bacterial invasion of commensal bacterial into a critical metabolic tissue, which receives ∽80% of its blood flow from the portal circulation connected to the gut. Gut mucin expression was not altered, but there may be an association with tight junction regulators in the ileum, given lower occludin levels in HFD-fed NOD2^−/−^ mice compared to HFD-fed WT mice. Our results are consistent with gut bacteria-derived factors regulating inflammation in metabolic tissues (Caesar *et al*, 2012), but an additional stress such as a HFD was required to reveal protective role of NOD2 regarding bacterial invasion and the associated metabolic defects. This bolsters the concept that PRRs (such as NODs) can control the inflammatory tone of metabolic tissues by acting as a relay between diet-induced signals to the gut microbiota and peripheral tissues, which must both adapt to a nutritional stress.

Defective NOD2 responses have established links to inflammatory bowel diseases (IBD) (Ogura *et al*, 2001). Our results show that dietary factors (such a fat intake) and genetic determinants (such as NOD2 frame shift mutations) should be considered as factors that contribute to potential links between metabolic disease and IBD. Interestingly, the bacterial and inflammatory mechanisms underlying gut inflammatory diseases and cancer and those contributing to insulin resistance appear to share commonalities given our results and those from others showing that the microbiota from NOD2^−/−^ mice predisposes mice to colitis and cancer (Couturier-Maillard *et al*, 2013). An intriguing possibility would be that an altered ecology of the microbiota dictates accumulation of bacterial components inside the host because a naive gut immune system allows new bacterial antigens to escape detection and proper elimination.

It is known from germ-free colonized experiments that transfer of the microbiota from obese to lean mice is sufficient to promote adiposity (Turnbaugh *et al*, 2006). Here, we have associated NOD2 with a microbial community that prevents excessive inflammation and insulin resistance during obesity. Similar to NOD2 up-regulation in adipose tissue during a HFD, transfer of the microbiota from HFD-fed NOD2^−/−^ mice alone preferentially increased NOD2 transcript levels in adipose tissue. This positions NOD2 as a metabolic tissue sensor that responds to bacterial cues. It is not yet clear if NOD2 deletion within a particular cell type is the critical link to glucose homeostasis. A HFD increased NOD2 in both adipocytes and hepatocytes. We also clearly show that a deletion of NOD2 within hematopoietic cells, an immune cell precursor population usually considered a key regulator of inflammation during diet-induced obesity, is not involved as primary mechanism for the control of glucose metabolism in response to a fat-enriched diet. Therefore, our data are consistent with a model where NOD2 sensing of PGN monitors resident gut microbiota and gut permeability and defects in this sensing system promote the translocation of specific bacteria or bacterial components toward metabolic tissues such as the liver and the adipose depots, which exacerbates metabolic inflammation, triggering insulin resistance, during a HFD.

Intriguingly, augmented obesity beyond that often seen in WT HFD-fed mice was not required for worse glucose tolerance in the absence of NOD2. Our results in F1 littermate experiments demonstrated that NOD2 contributes to glucose homeostasis independently of potential host or microbe genetic drift that may occur due to continual NOD2^−/−^ homozygous breeding. This is important since recent reports show minimal differences in the gut microbiome of littermate NOD2^+/+^ and NOD2^−/−^ mice in the absence of dietary stress (Robertson *et al*, 2013). Our results show that the dysbiosis in our NOD2^−/−^ mouse colony, when fed a HFD, is an independent factor contributing to insulin resistance. However, in our antibiotic and microbiota transfer experiments using non-littermate mice, we cannot discount familial transmission of the microbiota during breeding (Ubeda *et al*, 2012) rather than a direct role for NOD2-dictated immunity in our dysbiosis-dependent changes in metabolism.

In summary, NOD2 deletion contributes to insulin resistance during a HFD. An intact bacterial PGN-NOD2 sensing system, at least in part in non-hematopoietic cells, is required to prevent increased opportunity of bacterial colonization of the gut and bacterial translocation into metabolic tissues. Bacterial inputs from the gut alter glucose homeostasis, and this relationship is magnified in the absence of NOD2 in non-hematopoietic cells. Our results support the notion that specific bacterial PGN-mediated cues must be recognized by NOD2 to restrict bacterial invasion of metabolic tissues and prevent exacerbated metabolic inflammation. NOD2 sensing of bacterial PGN provides protection from metabolic defects and reinforces that immune proteins can control bacterial homeostasis, which can contribute to insulin resistance and diabetes during high-fat feeding and obesity.

## Materials and Methods

### Animal experiments

All procedures were approved by McMaster University Animal Ethics Review Board or ethical committee of the Rangueil Hospital. All NOD2^−/−^ mice originated from INSERM U786 (Paris, France) and have been backcrossed to at least the 10^th^ generation on a C57BL/6 background. Male WT and NOD2^−/−^ mice were on a HFD where ∽45% of calories are derived from fat (Research Diets, New Brunswick, NJ, USA: D12451) or a chow diet (∽5% fat) for 16 weeks. WT mice were from Charles River (Sherbrooke, Quebec, Canada or St Germain sur l'Arbresle, France) or in-house littermates, as indicated. Mice from a given condition were housed in at least 2 separate cages. Blood glucose and serum insulin measurements and glucose and insulin tolerance tests were done after 6 h of fasting, as described (Schertzer *et al*, 2009, 2011). Hyperinsulinemic euglycemic clamps were performed in conscious, 6-h fasted mice, after 16 weeks of HFD, as we have described (Galic *et al*, 2011; Jorgensen *et al*, 2012). Antibiotics (1.0 g/l ampicillin and 0.5 g/l neomycin) were provided in the drinking water and changed every 2 days for a period of 4 weeks. For the microbiota transfer experiment, germ-free C57BL/6 (8–10 weeks old) mice were obtained from the Farncombe Gnotobiotic Unit of McMaster University and were immediately and continually colonized by housing mice in week-old soiled litter from SPF C57BL/6 WT or NOD2^−/−^ donors (from the Central Animal Facility of McMaster University). These mice were housed using ventilated racks, and all mice were handled only in the level II biosafety hood to prevent bacterial contamination (Bercik *et al*, 2011).

### Bone marrow chimeras

Eight-week-old WT or NOD2^−/−^ mice were lethally irradiated (1,000 rad) and subsequently injected (i.v.) with 1 × 10^6^ bone marrow cells from WT or NOD2^−/−^ mice. After an 8-week rest, mice were placed on a 45% HFD for 16 weeks.

### Transcript detection, signaling, and immunohistochemistry

Total RNA was prepared from gonadal fat, liver, or tibialis anterior muscle using TRIzol, then DNase I-treated and reverse-transcribed according to the manufacturer's instructions (Invitrogen, Carlsbad, CA). Transcript levels of individual host genes were analyzed by quantitative PCR (qPCR) using the Rotorgene 6000 and assay on-demand TaqMan primer probes and kits, which were normalized to β-actin or RNA polymerase (RNAP), as described (Schertzer *et al*, 2011; Jorgensen *et al*, 2012). Immunoblotting was done in lysates prepared from postclamp tissues (muscle, adipose) and after acute injection of insulin (0.5 IU/kg) into the vena cava (liver), as we described previously (Galic *et al*, 2011; Hawley *et al*, 2012). Formalin-fixed adipose and liver tissues were cut in 8-μm sections by Pathology Laboratory Services at McMaster Children's Hospital. Immunohistochemical (IHC) detection of F4/80 was done using 10 μg/ml rat anti-mouse F4/80 antibody (AbD Serotec, Raleigh, NC, USA) and Vectastain ABC and DAB substrate (Vector Laboratories) and counterstained with Mayer's hematoxylin, as described in Galic *et al* (2011).

### Generation of the *E. coli* strain lacking *amiA* function

Δ*amiA E. coli* mutant was generated by transduction with bacteriophage P1. A P1 lysate of a *Shigella* mutant in the *amiA* gene (M90TΔamiA) was used to transfer the interrupted *amiA* region into a commensal mouse WT *E. coli* (Amar *et al*, 2011). Transductants were selected on the basis of kanamycin resistance and were analyzed by PCR with primers external to the mutation introduced: forward: 5′-cgttacctttttgcgggtta-3′ and reverse: 5′-aaatctggcgtgttcaggtc-3′.

### Quantitation of *E. coli* and bacterial DNA

Mice were sacrificed 2 h after feeding with 10^9^ cfu of WT or Δ*amiA* DsRed-*E. coli*. Translocation of *E. coli* from gut toward visceral adipose tissue was assessed by qPCR (viable and non-viable *E. coli*) and RT–qPCR (viable *E. coli*). Genomic DNA and total mRNA were isolated from visceral adipose tissue, using TRIzol reagent (Invitrogen, Cergy Pontoise, France). After homogenization of tissue in TRIzol, cells were mechanically disrupted by mini-beads vibrating at 30 Hz for 2 × 3 min on a Retsch Tissue Lyser II (Qiagen GmbH, Germany) with 200 mg of acid-washed glass beads (Ø < 106 nm; Sigma-Aldrich, France). Total mRNA and genomic DNA were then extracted according to the manufacturer's procedure (Invitrogen, Cergy pontoise, France). Primers specific for DsRed1 were as follows: DsRed forward, 5′-GGGAGCGCGTGATGAACTTCGAGGA-3′; DsRed reverse: 5′-CCTCGGTGCGCTCGTACTGCTCCAC-3′. The qPCR assay was performed on DNA or cDNA with a Stepone Plus Real-Time PCR System and Power SYBR Green PCR Master Mix (Applied Biosystems, California, USA). Data were analyzed by absolute quantitation using a standard curve of DNA or cDNA obtained from a pure culture of DsRed-*E. coli*. Mice were sacrificed 2 h after gavage with 10^9^ cfu of WT *E. coli* or Δ*amiA E. coli*. One centimeter of each intestinal segment was collected, emptied and opened, and rinsed in sterile water, and mucosa was collected by scraping. Visceral adipose tissue was harvested and weighed. Intestinal mucosa and adipose tissue were then homogenized in Luria–Broth (LB), plated onto ampicillin-supplemented (100 ng/ml) LB agar, and yellow (GFP-tagged) or pink (DsRed-tagged) colonies were enumerated after overnight incubation at 37°C. Two hours after bacterial gavage, a separate group of mice were sacrificed and adipose tissue was prepared as described in the Supplementary Methods to quantify GFP^+^ cells in the SVF.

### Isolation, preparation, and qPCR of endogenous liver and adipose bacterial DNA

Total bacterial DNA was extracted from snap-frozen liver and adipose tissue specimen using the QIAamp DNA mini stool kit (Qiagen, Courtaboeuf, France), which also included homogenization using a (≤ 106 μm diameter) bead-beating step (6,500 rpm, 3 × 30 s). The DNA was amplified by real-time PCR (Stepone+; Applied Biosystems) in optical grade 96-well plates. The PCR was performed in a total volume of 25 μl using the Power SYBR® Green PCR master mix (Applied Biosystems), containing 300 nM of each of the universal forward and reverse primers F-Bact1369 (5′- CGGTGAATACGTTCCCGG-3′) and R-Prok1492 (5′-GGACTACCAGGGTATCTAATCCTGTT-3′) (Sokol *et al*, 2008). The reaction conditions for amplification of DNA were 95°C for 10 min and 40 cycles of 95°C for 15 s and 60°C for 1 min. The amplification step was followed by a melting curve step according to the manufacturer's instructions (from 60°C to 95°C) to determine the specificity of the amplification product obtained. The amount of DNA amplified was compared with a purified 16S DNA from *E. coli* BL21 standard curve, obtained by real-time PCR from DNA dilutions ranging from 0.0001 to 1 ng/μl.

### Bacterial profiling by deep sequencing analysis of 16S rRNA with Illumina

The V3 region of the 16S rRNA gene was amplified by PCR, as previously described (Bartram *et al*, 2011). Briefly, triplicate PCRs were pooled and individual amplification was carried out for each sample, using 50 μl reaction mixture containing 25 pmol of each primer, a 200 μM concentration of each deoxynucleoside triphosphate (dNTP), 1.5 mM MgCl_2_, and 1 U Taq polymerase (Invitrogen, Burlington, ON). Illumina sequencing and preliminary analysis were carried out by the MOBIX-McMaster Genome Center (McMaster University). PCR products of the correct size were then purified by electrophoresis on a 2% agarose gel and recovered using a QIAquick gel extraction kit (Qiagen, Mississauga, Ontario, Canada). Custom, in-house Perl scripts were developed to process the sequences after Illumina sequencing (Whelan *et al*, 2014). Cutadapt was used to trim any over-read sequences (Martin, 2011), and paired-end sequences were aligned with PANDAseq (Masella *et al*, 2012). Mismatches and ambiguous base attributions in the assembly from specific set of paired-end sequences were discarded. Operational taxonomic units (OTUs) were picked using AbundantOTU+ and taxonomy-assigned using the Ribosomal Database Project (RDP) classifier against the Greengenes reference database (Ye, 2011). Calculations of within-community diversity (α-diversity) and between-community diversity (β-diversity) were run using QIIME (Caporaso *et al*, 2010). Sequencing characteristics for each sample are described in Supplementary Table S1.

### Statistical analysis

Previous experience and the magnitude of the experimental effect in preliminary experiments informed estimation of sample size in each experiment. No samples were excluded from analysis, expect in the experiments where body mass matching was done between the two different genotypes. For body mass matching, we only included data from mice in each genotype where the average body mass of each group was not different. We did not use animal randomization or blinding of investigators. Values are mean ± SEM, unless specified. Statistical tests were justified for each figure, as appropriate. Statistical significance was determined by unpaired *t*-test or ANOVA, and Tukey's post-hoc tests were used where appropriate (Prism 4-6, GraphPad Software, USA). *P* < 0.05 was considered significant.

## Supporting Information

Supplementary Information

Review Process File
