# Supplementary figures and images for: Defective NOD2 peptidoglycan sensing promotes diet-induced inflammation, dysbiosis, and insulin resistance
Source: EMBO Mol Med. 2015 Feb 9;7(3):259–74. doi: 10.15252/emmm.201404169 (PMC4364944; doi:10.15252/emmm.201404169)

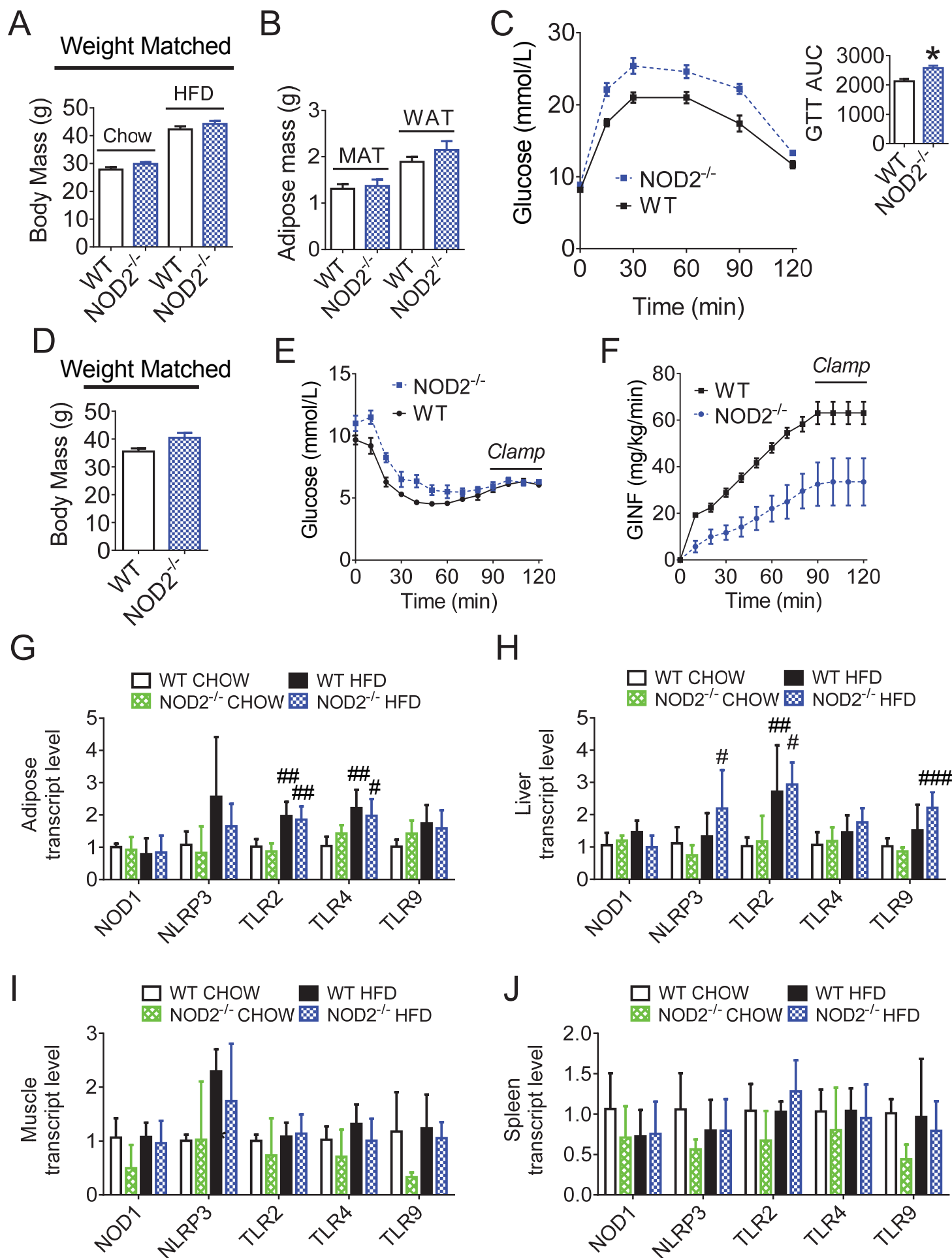

Fig S1

Supplement: Supplementary file 1 [file emmm0007-0259-sd1.pdf]

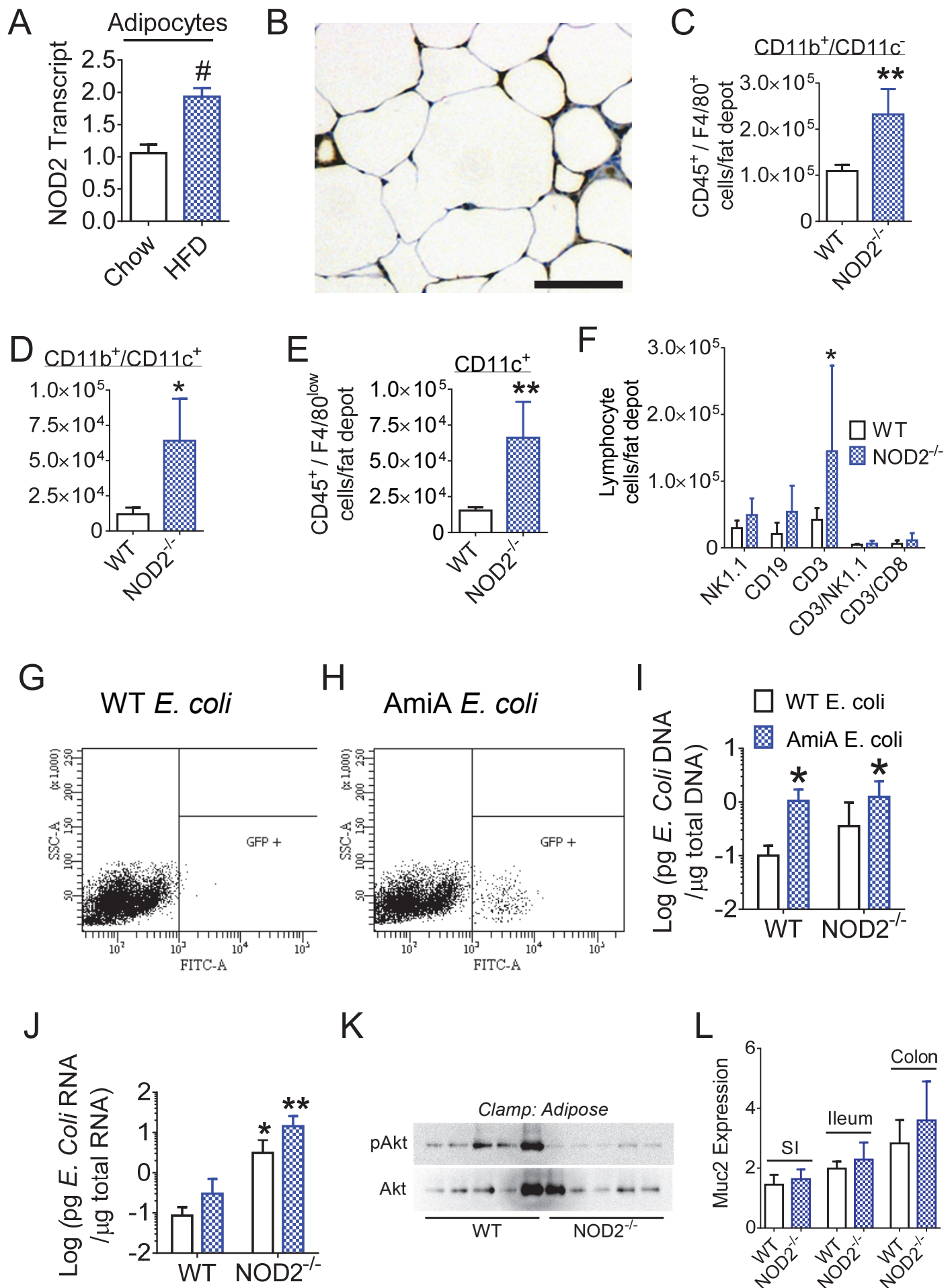

Fig S2

Supplement: Supplementary file 2 [file emmm0007-0259-sd2.pdf]

**A**

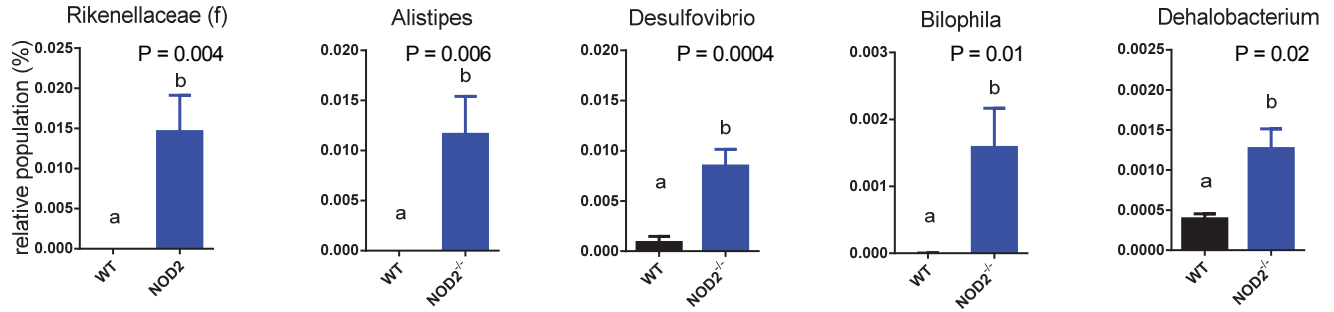

**B**

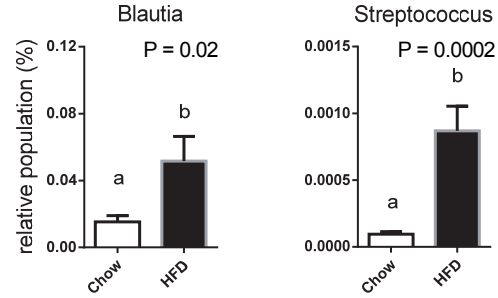

**C**

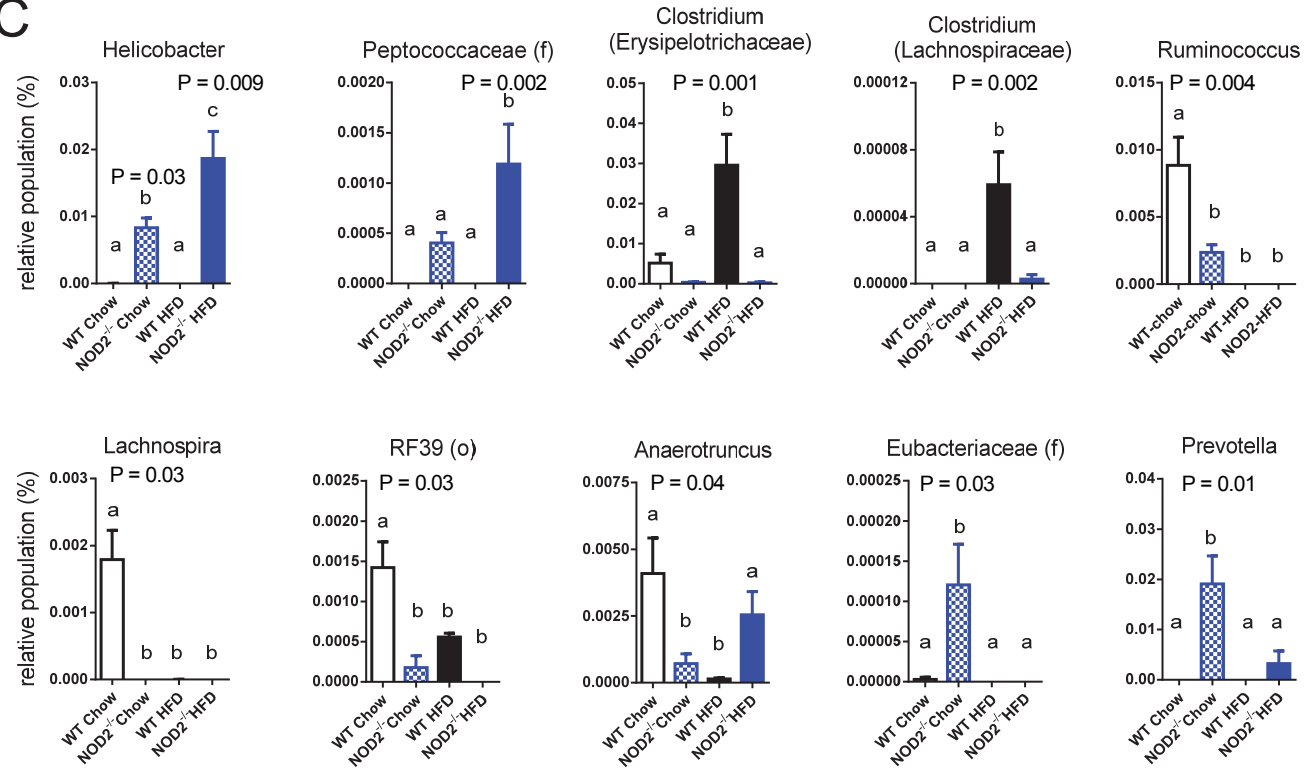

**Fig S3**

Supplement: Supplementary file 3 [file emmm0007-0259-sd3.pdf]

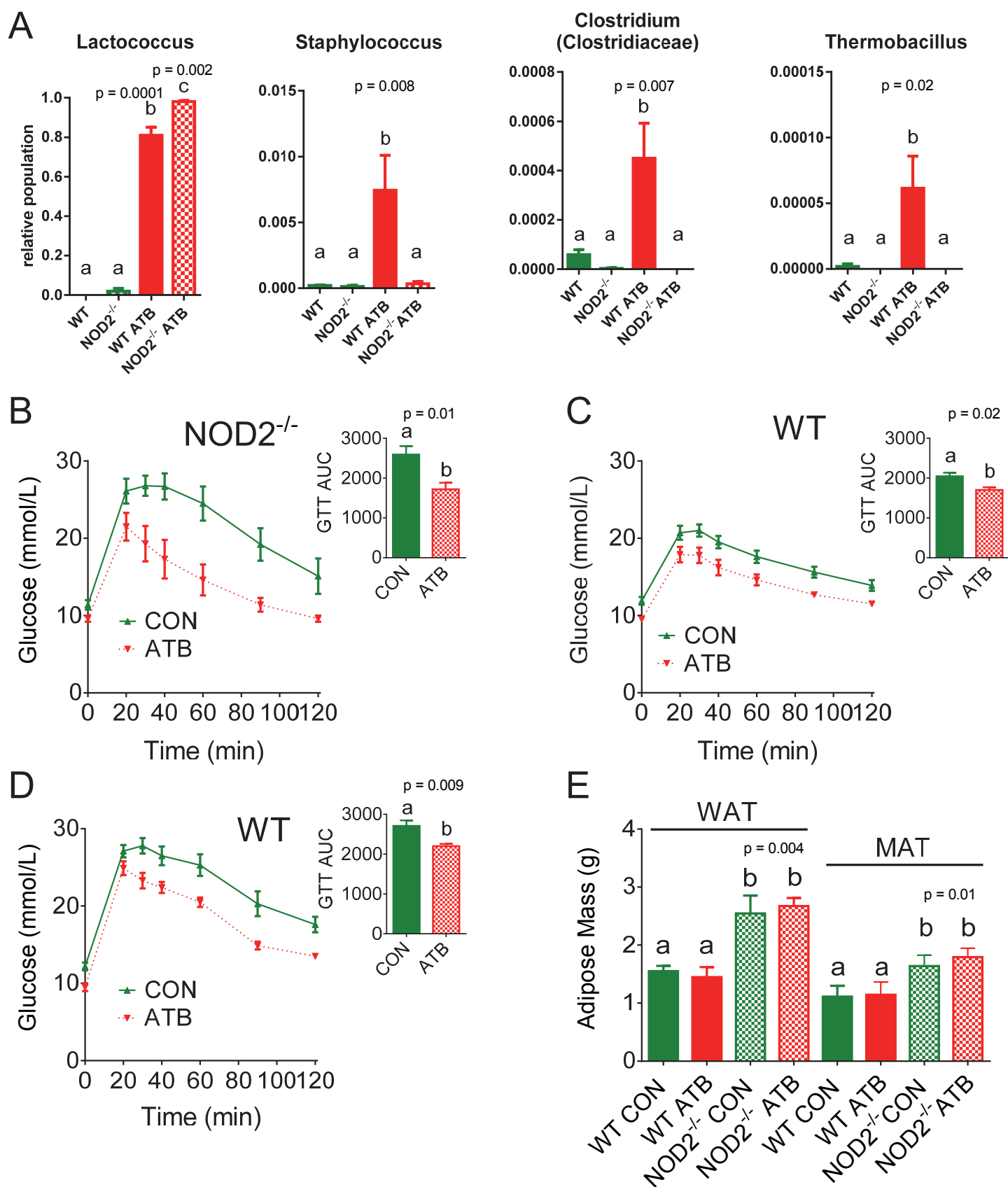

Fig S4

Supplement: Supplementary file 4 [file emmm0007-0259-sd4.pdf]

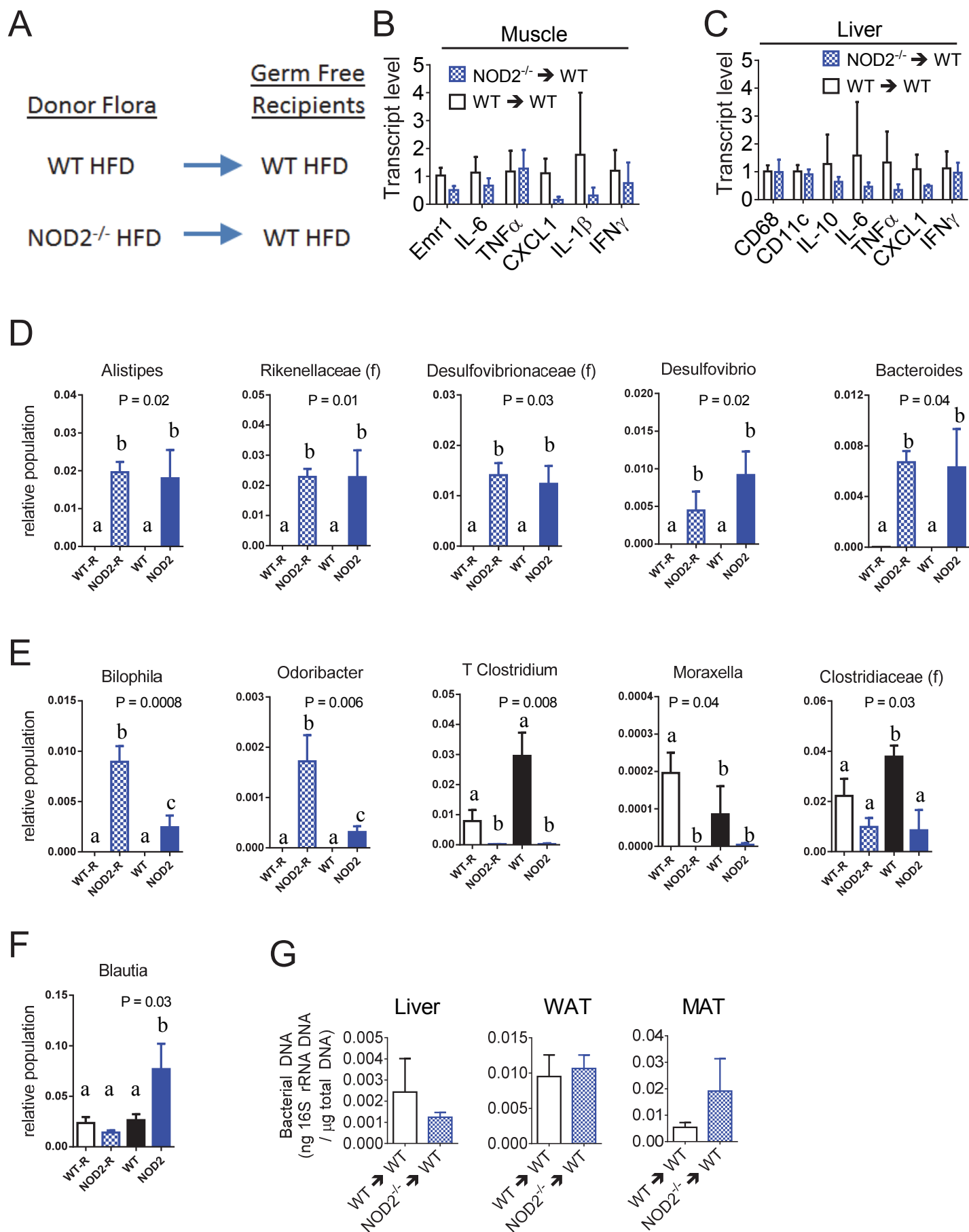

Fig S5

Supplement: Supplementary file 5 [file emmm0007-0259-sd5.pdf]
