# Supplementary material for: Defective NOD2 peptidoglycan sensing promotes diet-induced inflammation, dysbiosis, and insulin resistance
Source: EMBO Mol Med. 2015 Feb 9;7(3):259–74. doi: 10.15252/emmm.201404169 (PMC4364944; doi:10.15252/emmm.201404169)
Supplement: Supplementary file 6 [file emmm0007-0259-sd6.docx]

**Supplementary Information**

**Table of contents**

**Supplementary methods 2**

**Supplementary reference 3**

**Supplementary table 4**

**Supplementary figure legends 5**

**Supplemental methods.**

For experiments presented in panels D to G in Figure S2, WT and NOD2^-/-^ mice were fed a very HFD (70% Kcal from Fat, Safe, Augy, France) for 4 weeks to rapidly study the early onset of the disease before further metabolic complications ([Amar et al, 2011](#_ENREF_1)).

**Flow cytometry analyses**

Mice were sacrificed and adipose tissue was minced and digested (Sigma Aldrich, Saint Quentin Fallavier, France; 250 units collagenase / ml in PBS and 2 % bovine serum albumin (BSA)) for ~1 h. The suspension was filtered through a 250 μm sieve and centrifuged (400 g; 10 min; RT). The pellet containing the cells from the SVF was then resuspended and incubated for 10 min in erythrocyte-lysing buffer (155 mM NH4Cl, 5.7 mM K2HPO4 and 0.1 mM EDTA). After 37 μm filtration and centrifugation (400 g; 10 min; 4°C), cells were resuspended in PBS containing 2 mM EDTA and 0.5% BSA. The total number of cells was counted using trypan blue (Gibco, Courbevoie, France). 150 000 cells of the SVF were incubated for 25 min at 4°C with PerCP-conjugated antibodies (CD45, NK-1.1), APC-conjugated antibody (CD31, F4/80 and CD8), PE-Cy7 conjugated antibody CD19, APC-Cy7 conjugated antibody (CD3, Sca1) and respective isotype controls. Labeled cells were then washed in PBS, centrifuged (400 g; 10 min; 4°C) and then analyzed by flow cytometry using a FACSCalibur flow cytometer and Diva software (BD-Biosciences, Le Pont-de-Claix, France). The total number of each cell population present in the adipose tissue depot was calculated as a product of the percentage of each cell type determined by the flow cytometry analyses and the total number of SVF cells counted.

**Supplementary reference**

Amar J, Chabo C, Waget A, Klopp P, Vachoux C, Bermúdez-Humarán LG, Smirnova N, Bergé M, Sulpice T, Lahtinen S et al (2011) Intestinal mucosal adherence and translocation of commensal bacteria at the early onset of type 2 diabetes: molecular mechanisms and probiotic treatment. EMBO Molecular Medicine 3**:** 559-572

**Supplementary table.**

**Table 1. Supporting table from QIIME 16S rDNA sequencing analysis**

|  | Diet and NOD2 genotype (Fig 5) | Antibiotics and Microbiota Transfer (Fig 6 and 7) |
| --- | --- | --- |
| Number of Samples | 22 | 31 |
| Minimum Sequences Count | 57979 | 16372 |
| Maximum Sequences Count | 116375 | 126798 |
| Median Sequences Count | 89204 | 80786.5 |
| Normalized Sequences**^#^** | 57979 | 16372 |
| Number of 97% phylotypes (genus level-assignable) | 310 | 303 |

**^#^**The normalized number of sequences represents the number of sequences that each dataset (of a given sample) were normalized to by rarefaction to allow for intra-sample comparisons of the datasets.

**Supplementary figure legends.**

**Figure S1.**

Body mass (A) and gonadal white adipose tissue (WAT) and mesenteric adipose tissue (MAT) mass (B) of 24-25 week old weight-matched mice that were on a chow (WT, n = 11, NOD2^-/-^, n = 10) or HFD (WT, n = 9, NOD2^-/-^, n = 10) for 16 weeks. Blood glucose and the cumulative AUC during glucose tolerance tests (GTT; 1.5 g/kg *i.p.*) in HFD-fed WT (n = 6) and NOD2^-/-^ (N= 6) mice (C), *P = 0.03. Body mass (D), blood glucose levels (E) and glucose infusion rate (GINF) during the entire clamp protocol in weight-matched HFD-fed WT (n =4) and NOD2^-/-^ (n =3) mice. qPCR detection of NOD1, NLRP3, TLR2, TLR4 and TLR9 in gonadal adipose tissue (G), from 24-25 week old WT and NOD2^-/-^ mice after a chow (n = 5 in all tissues and both genotypes) or 16 week HFD (n = 11 in all tissues and both genotypes), ^#^P = 0.03, ^##^P = 0.001. qPCR detection of NOD1, NLRP3, TLR2, TLR4 and TLR9 in liver (H) tibialis anterior skeletal muscle (I) and spleen (J) from 24-25 week old WT and NOD2^-/-^ mice after a chow (n = 5 in all tissues and both genotypes) or 16 week HFD (n = 11 in all tissues and both genotypes), ^#^P = 0.02, ^##^P = 0.01, ^###^P = 0.0007 . *Significantly different from HFD-fed WT mice. ^#^Significant different from chow fed mice of the same genotype (p < 0.05).

**Figure S2.**

NOD2 transcript levels in primary adipocytes (A) isolated from WAT of chow-fed (n = 10) and HFD-fed (n = 5) WT mice, ^#^P = 0.001. IHC staining of F4/80^+^ cell in WAT (B). WT (n = 7) and NOD2^-/-^ (n = 4) mice fed a very HFD containing 70% Kcal from fat for 4 weeks and visceral adipose tissue SVF were analyzed by flow cytometry for: macrophage (CD45^+^/F4/80^+^) subsets (CD11b^+^/CD11c^-^, C) (CD11b^+^/CD11c^+^, D); dendritic-like cells (CD45^+^/F4/80^low^/CD11c^+^; E); and lymphocytes (F), including natural killer cells (NK1.1^+^), B lymphocytes (CD19^+^), total T cells (CD3^+^), NK T cells (CD3+/NK1.1^+^), cytotoxic, T cells (CD3^+^/CD8^+^), *P = 0.04, **P = 0.02. Representative side scatter versus GFP fluorescence FACS dot plots of the adipose tissue stromal vascular fraction (SVF) from WT mice 2 hours after administration of 10^9^ cfu WT GFP-*E. coli* (G) or *∆amiA* GFP-*E. coli* (H). Bacterial DNA (pg *E. coli* DNA/μg total DNA) in adipose tissue assessed by qPCR 2 hours after oral administration of 10^9^ cfu of WT or mutant (*∆AmiA*) DsRed labeled *E. coli* (n=5 in all groups) (I), *P = 0.03. Bacterial RNA concentration (pg *E. coli* RNA/μg total RNA) (J) in adipose tissue assessed by qPCR 2 hours after oral administration of 10^9^ cfu of WT or mutant (*∆AmiA*) DsRed labeled *E. coli* (n=5 in all groups), *P = 0.05, **P = 0.002. Representative immunoblot of pAkt and total Akt in adipose tissue after hyperinsulinemic euglycemic clamps in WT (n =5) and NOD2^-/-^ (n = 5) mice (K). ^#^Significantly different from chow fed WT mice, *Significantly different from WT HFD-fed mice or WT *E. Coli* given to WT mice (p < 0.05).

**Figure S3.**

Illumina sequencing and bacterial profiling of the cecum in chow and 16 week HFD-fed WT and NOD2^-/-^ mice showed genera regulated by genotype (A), diet (B) and the interaction of genotype and diet (C). n = 6 for WT and NOD2^-/-^ chow fed mice. n = 5 for WT and NOD2^-/-^ HFD-fed mice. Conditions with different letters denote a statistical difference (P <0.05, n>5/group), where the minimal statistical values for all comparisons are shown on the appropriate panel.

**Figure S4.**

HFD-fed WT and NOD2^-/-^ mice were tested without (CON) or with antibiotics (ATB) (1.0 g/L ampicillin, 0.5 g/L neomycin) in the drinking water for the final 4 weeks of a 16 week HFD. Illumina sequencing and bacterial profiling of the cecum in 16 week HFD-fed WT and NOD2^-/-^ mice without (CON) or with antibiotics (ATB) in the drinking water for the final 4 weeks of the HFD showed genera specifically regulated by the interaction of genotype and antibiotics (A) (n = 5 mice for all groups). Effect of ATB on blood glucose and the cumulative AUC during GTTs in NOD2^-/-^ (B) and WT (C) HFD-fed mice where the same absolute glucose load was delivered (1.5 g/kg *i.p.* D-glucose) (n = 5 mice for all groups). Glucose tolerance was also assessed in HFD-fed WT mice where the glucose load was carefully titrated to match the response in HFD-fed NOD2^-/-^ mice (1.8 g/kg *i.p.* D-glucose) (D) (n = 5 mice for all groups). Gonadal white adipose tissue (WAT) mass and mesenteric adipose tissue (MAT) mass in (CON) or antibiotics (ATB) treated HFD-fed WT and NOD2^-/-^ mice (E). Conditions with different letters denote a statistically difference, where the minimal statistical values for all comparisons are shown on the appropriate panel.

**Figure S5.**

Schematic of the strategy used to transfer the microbiota from donor WT and NOD2^-/-^ mice fed a high fat diet (HFD) for 16 weeks to 8-10 week old recipient WT germ-free mice (A). Immune cell and inflammation markers in tibialis anterior muscle (B) and liver (C), 5 weeks after microbiota reconstitution from WT donor into previously germ-free WT mice (n =5) and NOD2^-/-^ donor into previously germ-free WT mice (n =4). Illumina sequencing and bacterial profiling of the cecum in HFD-fed WT and NOD2^-/-^ donor and previously germ free WT recipient mice showed genera specifically transferred between NOD2^-/-^ donor and recipient mice to comparable levels (D) (n = 5 for all groups of mice). Bacterial profiling also showed genera specifically transferred between NOD2^-/-^ donor and recipient mice albeit at a different prevalence in the cecum and specific genera transferred between the WT donor and recipient mice (E) (n = 5 for all groups of mice). Some genera were different in NOD2^-/-^ donors compared to other conditions (F) (n = 5 for all groups of mice). Quantification of bacterial DNA in the liver, WAT and MAT 5 weeks after microbiota reconstitution from WT donor into previously germ-free WT mice (n =5) and NOD2^-/-^ donor into previously germ-free WT mice (n =4). (G). Conditions with different letters denote a statistical difference, where the minimal statistical values for all comparisons are shown on the appropriate panel.
