# Supplementary material for: Defective NOD2 peptidoglycan sensing promotes diet-induced inflammation, dysbiosis, and insulin resistance
Source: EMBO Mol Med. 2015 Feb 9;7(3):259–74. doi: 10.15252/emmm.201404169 (PMC4364944; doi:10.15252/emmm.201404169)
Supplement: Supplementary file 7 [file emmm0007-0259-sd7.pdf]

Defective NOD2 peptidoglycan sensing promotes diet-induced inflammation, dysbiosis and insulin resistance

Emmanuel Denou, Karine Lolmède, Lucile Garidou, Celine Pomie, Chantal Chabo, Trevor C. Lau, Morgan D. Fullerton, Giulia Nigro, Alexia Zakaroff-Girard, Elodie Luche, Céline Garret, Matteo Serino, Jacques Amar, Michael Courtney, Joseph F. Cavallari, Brandyn D. Henriksbo, Nicole G. Barra, Kevin P. Foley, Joseph B. McPhee, Brittany M. Duggan, Hayley M. OíNeill, Amanda J. Lee, Philippe Sansonetti, Ali A. Ashkar, Waliul I. Khan, Michael G. Surette, Anne Bouloumié, Gregory R. Steinberg, Rémy Burcelin, Jonathan D. Schertzer

Corresponding author: Jonathan Schertzer, McMaster University

Review timeline:

Submission date:	14 April 2014
Editorial Decision:	15 May 2014
Revision received:	16 December 2014
Editorial Decision:	09 January 2015
Revision received:	12 January 2015
Accepted:	16 January 2015

Transaction Report:

(Note: With the exception of the correction of typographical or spelling errors that could be a source of ambiguity, letters and reports are not edited. The original formatting of letters and referee reports may not be reflected in this compilation.)

Editor: Céline Carret

1st Editorial Decision 15 May 2014

Thank you for the submission of your manuscript to EMBO Molecular Medicine. We have now heard back from the three referees whom we asked to evaluate your manuscript. Although the referees find the study to be of potential interest, they also raise a number of concerns that need to be addressed in the next version of your manuscript.

As you will see, while referee 1 is supportive of publication as is, the other two referees are much more reserved and raise serious issues regarding the experimental model used; however, both referees suggest performing additional work that would provide mechanistic insights and somehow overcome the limitations of the model. In addition, referee 2 is concerned about the reproducibility of the data and I would strongly encourage you to make clear how many times each experiment was reproduced (biological replicates) and provide "n" in each case. This referee also noted that figure 5 and figure 7 use the same data but this is not made clear in the figure legend and main text. Please carefully modify the study that it becomes obvious that both experiments are in fact a single one displayed twice.

We would welcome the submission of a revised version for further consideration and depending on the nature of the revisions, this may be sent back to the referees for another round of review.

Please note that it is EMBO Molecular Medicine policy to allow a single round of revision in order

to avoid the delayed publication of research findings. Consequently, acceptance or rejection of the manuscript will depend on the completeness of your responses included in the next version of the manuscript.

EMBO Molecular Medicine has a "scooping protection" policy, whereby similar findings that are published by others during review or revision are not a criterion for rejection.

Should you decide to submit a revised version, I do ask that you get in touch after three months if you have not completed it, to update us on the status.

Please also contact us as soon as possible if similar work is published elsewhere. If other work is published we may not be able to extend the revision period beyond three months.

I look forward to seeing a revised form of your manuscript as soon as possible.

***** Reviewer's comments *****

Referee #1 (Remarks):

The manuscript by Denou et al. deals with the influence of NOD2 peptidoglycan sensing on diet induced insulin resistance and that this factor controls gut microbial colonizaton.
The authors use well suited methods, but minor revisions should be made before publication.
It would be nice if the authors could discuss more the different results they obtained before on muscle insulin resistance and NOD2 and the influence of whole body ko of NOD2 under HFD.

page 5: line 5-6: revise sentence: "NOD2 immunity may also to contribute to...

page 5: line: line 23: do not judge on methods: remove: " The gold standard measure of insulin resistance"

page 16: line 7: correct "tibalis" to "tibialis"

page 18: line 6 and 7: include a space between numbers and units

page 18: line 20: correct "MgCl2"

page 30: line 6, page 32: line 1, kine 15, page 33 line 5: correct grammar on "denotes"

Fig 2F: please show western blot
Fig 4F: please show also graph of PTT curves
Fig 4I: please show western blot
Fig 6I: Please show graph of GTT curves

Referee #2 (Remarks):

In this manuscript, Denou et al identified the link between the PGF sensor NOD2 and HFD-induced insulin resistance. HFD-fed NOD2-/- mice showed exacerbated glucose tolerance and higher HOMA-IR compared to wild type mice. This phenotype results from adipose tissue inflammation induced by enhanced bacterial translocation and altered gut bacterial communities. Transfer of gut microbiota from HFD-fed NOD2-/- mice predisposes germ-free mice to the metabolic syndrome. The manuscript is well written and shows an interesting phenotype with a mechanistic insight. I however have several concerns as described below.

Specific comments:

1. Enhanced translocation of gut microbiota in HFD-fed NOD2-deificient mice suggests gut barrier dysfunction. This possibility is discussed, but not directly addressed. Analyses of mucus layer and tight junction as well as gut immune balance would strengthen this conclusion.

2. NOD2 is constitutively expressed by myeloid cells and Paneth cells. Which cell type contributes to the NOD2-dependent peptideglycan sensing system to limit accumulation of bacteria? Because NOD2-deficient mice have been backcrossed onto B6 genetic background, bone marrow chimeric mice can be established to answer this question.

3. Microbiome data on the HFD groups in Fig. 5B are obviously identical to those on the donor groups in Fig. 7B. If so, it is fair to mention this fact on the figure legend. Further, the phylum labels are deferent on the two panels; the label for Verrucomicrobia is missing in Fig. 5B and the positions of columns for Tenericutes and Actinobacteria are opposite between Fig.5 and Fig. 7. I am concerned about reproducibility of the animal experiments throughout the manuscript. How many times did the authors perform each experiment? If the authors have never repeated, it is highly recommended to confirm reproducibility by performing at least the main part of experiments again. Then, how many times they repeated experiments should be described in Figure legends.

4. In Fig. 2B, it is difficult to identify macrophage infiltration in the adipose tissue. Embedded higher magnification pictures would be informative for readers. In Figure 2C, the expression levels of Erm1 and CD11c are only marginally upregulated in HFD-fed NOD2-/- mice compared to WT mice. Flow cytometric analysis is necessary to rigorously confirm the enhanced infiltration of macrophages and dendritic cells into adipose tissue.

Minor issues:
In Shannon index and similarity index data, there are no explanation for a, b, c, d and e.
In Fig. 1A, the Y axis label should be Body Mass.
In Fig. 7C, why are there two groups for WT-R?
Fig. S5 is considered typo of E3.

Referee #3 (Comments on Novelty/Model System):

Inadequate in the sense that total body knockout mice were used.

Referee #3 (Remarks):

Denou et al. investigated the role of the NOD2-PGN sensing system on obesity-associated metabolic dysfunction. They report worsening of obesity-associated glucose and insulin tolerance in NOD2 knockout mice. In addition, they provide evidence that the NOD2-PGN system regulates gut mucosal bacterial colonization as well as bacteria translocation to adipose tissue and the liver where the latter may trigger metabolic inflammation and insulin resistance. Moreover, they demonstrate that altered gut microbiota composition contributes to worsened metabolic phenotype in NOD2 knockout mice and is transmissible to germ-free mice.
Overall, the presented study is carefully conducted and interesting. The finding of improved adipose tissue inflammation in NOD2 deficient mice is unexpected and the underlying mechanism is unclear.

Major comments
1) The main limitation of the present study is the use of whole body knockout mice. The reported phenotype is clearly interesting. However, it might be solely explained by the fact that NOD2 regulates gut microbiota and/or gut permeability (which was shown before), e.g. the observed metabolic phenotype is just the result of increased translocation of different bacterial species. Of course, generating tissue-specific knockout mice is beyond the scope of the present study. However,

additional experiments should provide further insights. For example, NOD2 expression and/or protein levels may be determined in adipocytes and the SVF of adipose tissue harvested from chow- and HFD-fed mice. Similarly, NOD2 expression may be analyzed in hepatocytes and Kupffer cells of these mice. If NOD2 is mainly up-regulated in myeloid cells authors should perform adoptive bone marrow transfer of NOD2-/- into WT mice in order to generate hematopoietic NOD2-deficient mice.
2) Fig. 7 depicts higher fasting blood glucose levels and HOMA-IR index in germ-free WT mice receiving gut microbiota from HFD-fed NOD2-/- mice indicating exacerbated insulin resistance. Since this is a key finding of the present study and since the potential value of HOMA-IR to assess insulin resistance in mice is controversially discussed glucose as well as insulin tolerance tests should be performed in these mice.
3) It is several times stated (including abstract, results and discussion sections) that NOD2 detection of PGN protects against HFD-induced insulin resistance, adipose tissue inflammation etc. However, in the present study, a loss-of-function rather than a gain-of-function model was used. Thus, the authors should claim that ablation of NOD2 detection of PGN aggravates HFD-induced insulin resistance, adipose tissue inflammation etc. and that NOD2 may play a protective role in the development of obesity-associated insulin resistance and inflammation. Furthermore, these results may suggest that forced NOD2 expression/NOD2 binding suppress the production of pro-inflammatory cytokines. However, previous studies including their own have suggested the opposite. What would be the authors' explanation for such apparent discrepancy.
4) Fig E4 depicts increased WAT mass in NOD2-/- mice compared to WT mice. Was such increase/difference observed for all depots e.g. perigonadal, inguinal, mesenteric, etc. or are NOD2 mice prone to mainly increase fat mass at visceral sites. Such analysis should be presented in Fig. 1.

Minor comments
1) 2nd sentence of 1st paragraph/introduction: increased neutrophil infiltration of adipose tissue in HFD-fed was first shown by Elgazar-Carmon V et al. (J Lipid Res 2008). This article needs to be cited here.
2) Fig. 1 E: Please provide time course for glucose infusion rate and glucose levels during the whole clamp period in a supplementary figure.
3) Fig. 2F and 4I: Please show representative Western Blots for pAkt and pFOXO1.
4) Fig. 4E: The difference in liver lipids between the genotypes is convincing, especially when considering the TAG-data in Fig. 4F. However, one would expect that mice fed a HFD for 16 weeks would show some degree of hepatic steatosis, i.e. the presence of lipid droplets on H&E staining. Therefore the chosen microscopic image for control HFD-fed mice does not seem to be representative.

1st Revision - authors' response 16 December 2014

Editorial comments:

I would strongly encourage you to make clear how many times each experiment was reproduced (biological replicates) and provide "n" in each case. This referee also noted that figure 5 and figure 7 use the same data but this is not made clear in the figure legend and main text. Please careful modify the study that it becomes obvious that both experiments are in fact a single one displayed twice.

Response:

We have indicated the biological replicates as “n” for each experiment in the figure legends, as suggested by the reviewers and editorial comments. It is noteworthy that we have reproduced the major findings in many different cohorts of mice across 3 labs in Canada and France.

We apologize for not making clear that the microbiome from donor WT and $NOD2^{-/-}$ mice were analysed in 2 separate ways. We have removed the redundant data presented in the final figure (Figure 7) and made it very clear that the same mice were used in specific parts of Figure 5 analysis

(of all conditions) and as "donor mice" in of Figure 6. As an example, we have highlighted this in the calculation of the similarity index for reconstitution efficiency. On page 11 of the revised manuscript we state "The donor mice used in this experiment were the same HFD-fed WT and NOD2-/- mice from Fig 5, which allowed us to directly compare the microbiome of all conditions (Fig 6A, 7A, 7B)."

Reviewer #1:

The authors use well suited methods, but minor revisions should be made before publication.

It would be nice if the authors could discuss more the different results they obtained before on muscle insulin resistance and NOD2 and the influence of whole body ko of NOD2 under HFD.

Response:

Thank you. We have made all of these text changes and included all of the relevant data the revised manuscript -as outlined below.

page 5: line 5-6: revise sentence: "NOD2 immunity may also to contribute to...

"…contribute to gut microbial homeostasis, but …"

page 5: line: line 23: do not judge on methods: remove: " The gold standard measure of insulin resistance"

Removed

page 16: line 7: correct "tibalis" to "tibialis"

Corrected

page 18: line 6 and 7: include a space between numbers and units

Spaces included

page 18: line 20: correct "MgCl2"

Corrected to $MgCl_2$

page 30: line 6, page 32: line 1, kine 15, page 33 line 5: correct grammar on "denotes"

Corrected all to "denote"

Fig 2F: please show western blot

This data is in Fig E2

Fig 4F: please show also graph of PTT curves

This data is in Figure 4

Fig 4I: please show western blot

This data is in Figure 4

Fig 6I: Please show graph of GTT curves

This data is in Figure E4

Reviewer #2:

The manuscript is well written and shows an interesting phenotype with a mechanistic insight. I however have several concerns as described below.

© EMBO

1. Enhanced translocation of gut microbiota in HFD-fed NOD2-deificient mice suggests gut barrier dysfunction. This possibility is discussed, but not directly addressed. Analyses of mucus layer and tight junction as well as gut immune balance would strengthen this conclusion.

Response:

Thank you for the suggestion. We have now directly addressed any potential barrier dysfunction in vivo by measuring permeability of FITC-labelled dextran after oral gavage in new experiments. This has been done in new cohorts of all the different groups of mice. This *in vivo* test showed that a high fat diet (HFD) increased gut permeability, but NOD2 genotype did not change gut permeability to labelled dextran under either dietary condition.

We agree that this was an important experiment since assessment of a permeability defect provides important information regarding the mechanism of the increased abundance of bacteria or bacterial components or bacterial DNA observed in the liver of HFD-fed-NOD2-null mice. Our new data argues against an overt deficiency in gut permeability, but rather specificity for increased bacteria or bacterial component translocation when PGN sensing is compromised and in particular when NOD2-/- mice are fed a HFD.

As suggested by the reviewer, we have also assessed tight junction markers in the various gut segments of all mice. Our new results show that transcript levels of TJP-1 (ZO-1) is not influenced by diet or NOD2 genotype. Another marker of tight junction, occludin was not influenced by diet or genotype in the small intestine or colon. However, occludin was increased by a HFD in the ileum in WT mice, but not HFD-fed NOD2 mice. This was an interesting observation given that it shows a diet-genotype interaction which tracks with metabolic problems and inflammation. We have included some discussion on this in context with our bacterial translocation assays. As further suggested by the reviewer we have included new analysis of mucin transcripts involved in any potential differences in the regulation of mucus layer. Mucin1, 2, 3, 4, and 6 were not altered by diet or genotype in any gut segment analysed. Mucin6 was not reliably detected. These transcript data coupled with our new data showing that ONLY diet (not NOD2) regulated dextran permeability, we believe that we have alleviated any concern of overt barrier dysfunction elicited by lacking NOD2.

This new data is complementary to our existing data that directly addressed gut barrier function in WT and NOD2-/- mice using a functional assay with live bacteria. Fig 2 shows that NOD2-/- mice have more live bacteria in the adipose tissue after oral delivery (gavage) of the bacteria –and the only route this could take is through the gut. Fig E2 also supports this by measuring bacterial DNA and RNA that specifically detects only the tagged, gavaged bacteria. We have also shown the importance of peptidoglycan structure in these bacteria subverting the gut barrier. Fig 2 and E2 shows also showed that live GFP-expressing bacteria are inside a stromal vascular cell in the adipose tissue (i.e. passed through the gut).

We believe that it is most important that we showed increased bacterial DNA in the liver of HFD-fed NOD2-deficient mice compared to HFD-WT mice using the same obesity/dietary model that matches our metabolic parameters, including insulin resistance. The source of these bacteria in the liver is likely the gut (microbiota) given the anatomy of the portal circulation and direct route to the liver.

It is very difficult to capture gut barrier "function" using markers of "immune balance" and it is not entirely clear what is suggested as a measure by the reviewer. We have published on the potential route of diet-induced changes in bacteria penetrating through the gut (Amar, EMBO Mol Med, 2011) and the cause or consequence of any potential changes in gut immunity are not clear in relation to NOD2 sensing. We do appreciate the comments on gut barrier function, which we have now assessed after gavaging FITC-dextran and using markers of tight junctions.

2. NOD2 is constitutively expressed by myeloid cells and Paneth cells. Which cell type contributes to the NOD2-dependent peptideglycan sensing system to limit accumulation of bacteria? Because NOD2-deficient mice have been backcrossed onto B6 genetic background, bone marrow chimeric mice can be established to answer this question.

Response: This important issue was also raised by another reviewer. Combining these reviewer comments, it was also noted that NOD2 transcripts should be measured in hematopoietic and non-hematopoietic cells in chow versus HFD-fed animals. We completed both of the requested experiments by placing bone marrow transfer mice on a HFD and by measuring NOD2 transcripts in immune and metabolic cell populations. This was a good idea because we have previously shown (Schertzer et al., Diabetes 2011) that NOD2 is expressed in metabolic cells and immune cells.

First, in new cohorts of mice, we freshly separated the adipocytes from the stromal vascular fraction in adipose tissue and hepatocytes from non-hepatocytes (immune and vascular cells) in the liver. Our results show that NOD2 is up-regulated during a HFD in both metabolic cells types (adipocytes and hepatocytes).

Nevertheless, we made the chimeric mice (in both Canada and France), an experiment that took approximately 9 months to complete. Our results show that deletion of NOD2 from the non-hematopoietic compartment is the key driver of changes in glucose homeostasis. This is clear from increased glucose tolerance (AUC during a GTT) and increased HOMA-IR in NOD2-/- mice that received WT bone marrow, when directly compared to WT -> WT or even WT mice that were reconstituted with NOD2 bone marrow ($NOD2^{-/-}$ -> WT). This new data is in Fig 1. As suspected, a comprehensive tissue-specific deletion strategy that encompasses epithelial cells in the gut, adipocytes and hepatocytes or pancreatic cells is required to fully solve this issue. As noted by Reviewer #3, this is beyond the “beyond the scope of the present study”.

3. Microbiome data on the HFD groups in Fig. 5B are obviously identical to those on the donor groups in Fig. 7B. If so, it is fair to mention this fact on the figure legend. Further, the phylum labels are deferent on the two panels; the label for Verrucomicrobia is missing in Fig. 5B and the positions of columns for Tenericutes and Actinobacteria are opposite between Fig.5 and Fig. 7.

Response: Thank you for noting the redundant data. It was not our intention to suggest this was separate data. The “donor” mice, in figure 7 were in fact the same mice as WT-HFD and $NOD2^{-/-}$-HFD mice used in Figure 5. This was done by design since it allowed us to analyse all of this data at the same time to define the effects of diet, genotype, antibiotics and reconstitution efficiency. This is most evident by all of these groups of mice presented on a single PCoA graph in Figure 6. According to the reviewers comments and note from the editor, we have removed the redundant bar graphs for “donors” in figure 7 and made very clear, that similarity calculations and sequence analysis (i.e. cluster tree) between donors and recipient mice are based on donor mice that are, in fact, the same mice used in Figure 5. The phylum labelling in Figure 7 is slightly different compared to Fig 5 in order to keep in rank order the labelling of phylum abundance. We hope that removal of the redundant “donor” bar graphs has clarified this data.

I am concerned about reproducibility of the animal experiments throughout the manuscript. How many times did the authors perform each experiment? If the authors have never repeated, it is highly recommended to confirm reproducibility by performing at least the main part of experiments again. Then, how many times they repeated experiments should be described in Figure legends.

Response: We apologize for not making clear the number of experiments for each variable. We have highlighted the number of experiments in each Figure Legend.

We have repeated the major metabolic phenotypes many times in several different cohorts of mice spanning a 4 year period. We are pleased that three different labs have produced congruent results. As one example, the increased insulin resistance/intolerance in HFD-fed NOD2$^{-/-}$ mice was evident in more than 4 separate cohorts (n > 10) of mice when compared to HFD-fed WT mice. Further, hyperinsulinemic-euglycemic clamps are always done in a different cohort of mice, separately from glucose or insulin tolerance testing (ITTs). These experiments spanned many years and different cohorts of mice and we believe it is a significant strength of this paper that 3 labs across France and Canada have produced concordant results using separate mouse colonies and unique analysis tools. Our findings are even consistent across different dietary models used in the labs working on this project. For example, as outlined in the response to comment #4 below, similar markers of adipose tissue immune cells are increased when detected by flow cytometry after 4 weeks on a 70% HFD (completed in France; new data added to Figure E2) compared to qPCR after 16 weeks on a 45% HFD (completed in Canada; Fig 2). Finally, we have now conducted the bone marrow transplant studies that were requested by 2 reviewers and they have produced concordant results.

4. In Fig. 2B, it is difficult to identify macrophage infiltration in the adipose tissue. Embedded higher magnification pictures would be informative for readers. In Figure 2C, the expression levels of Erm1 and CD11c are only marginally upregulated in HFD-fed NOD2-/- mice compared to WT mice. Flow cytometric analysis is necessary to rigorously confirm the enhanced infiltration of macrophages and dendritic cells into adipose tissue.

We have provided a higher magnification and representative IHC image demonstrating the localization of macrophages as determined by f4/80 IHC in the revised (supplementary) Figure 2E. The expression levels of Emr1 and CD11c were significantly different. As requested by the reviewer we have further characterized these immune cells residing in adipose tissue using flow cytometry, which now shows concordant results. As proof of principle -we have included flow cytometry data from WT and NOD2$^{-/-}$ mice on a 70% HFD for 4 weeks. This new data in the revised figure E2 shows increased abundance of the immune cells with the same markers and characteristics (compared to qPCR) in the adipose tissue. The flow cytometry data has confirmed that macrophage-like (F4/80+) and dendritic-like cells (F4/80 low, cd11c+) are increased in HFD-fed NOD2$^{-/-}$ mice. We also show that among lymphocytes, there is a selective expansion of CD3 cells in the adipose tissue of HFD-fed NOD2$^{-/-}$ mice.

Minor issues:

In Shannon index and similarity index data, there are no explanation for a, b, c, d and e.

Response: We apologize for not making the notation for statistical significance clear for the Shannon index in Figure 7. We have now denoted in the figure legend that different letters equate to statistically significant difference between those groups. It is extremely cumbersome to use symbols or joining lines in this data set.

In Fig. 1A, the Y axis label should be Body Mass.

Response: Revised to Body Mass

In Fig. 7C, why are there two groups for WT-R?

Response: We apologize that this redundant comparison data was confusing. We have removed the redundant WT-R (on the right side of graph) from 7C

Fig. S5 is considered typo of E3.

Response: corrected to E3

Reviewer #3:

1) The main limitation of the present study is the use of whole body knockout mice. The reported phenotype is clearly interesting. However, it might be solely explained by the fact that NOD2 regulates gut microbiota and/or gut permeability (which was shown before), e.g. the observed metabolic phenotype is just the result of increased translocation of different bacterial species. Of course, generating tissue-specific knockout mice is beyond the scope of the present study. However, additional experiments should provide further insights. For example, NOD2 expression and/or protein levels may be determined in adipocytes and the SVF of adipose tissue harvested from chow- and HFD-fed mice. Similarly, NOD2 expression may be analyzed in hepatocytes and Kupffer cells of these mice. If NOD2 is mainly up-regulated in myeloid cells authors should perform adoptive bone marrow transfer of NOD2-/- into WT mice in order to generate hematopoietic NOD2-deficient mice.

Response: This important issue was also raised by another reviewer. Here, the reviewer has first suggested that NOD2 transcripts should be measured in hematopoietic and non-hematopoietic cells in chow versus HFD-fed animals, which is an excellent suggestion.

We have conducted new experiments in new cohorts of mice, where we freshly separated the adipocytes from adipose tissue and hepatocytes from non-hepatocytes (immune and vascular cells) in the liver from both chow and HFD-fed mice. Our results show that NOD2 is up-regulated during a HFD in both types of metabolic cells types (adipocytes and hepatocytes) within adipose and liver tissue. This is consistent with our previous results (Schertzer et al., Diabetes 2011) showing that NOD2 is expressed in metabolic cells (and immune cells), but adds important information in that the metabolic cells appear to be a point of regulation during obesity or a HFD for NOD2.

Although this reviewer suggested we make chimeric mice only if "mainly up-regulated in myeloid cells", we still made the chimeric mice given that this was suggested by 2 independent reviewers and editorial comments. We generated mice that had NOD2 deleted in the hematopoietic and non-hematopoietic compartments and placed these mice on a HFD for 16 weeks (in both Canada and France), an experiment took that approximately 9 months to complete. Our results show that deletion of NOD2 from the non-hematopoietic compartment is the key driver of changes in glucose homeostasis. This result is clear from increased glucose tolerance (AUC during a GTT) and increased HOMA-IR in HFD-fed NOD2$^{-/-}$ mice that received WT bone marrow, when directly compared to WT -> WT or even WT mice that were reconstituted with NOD2 bone marrow (NOD2$^{-/-}$ -> WT). This data is in Fig 1. As suspected, a comprehensive tissue-specific deletion strategy that encompasses epithelial cells in the gut, adipocytes and hepatocytes is required to fully solve this issue. As noted by this reviewer, this is beyond the "beyond the scope of the present study".

2) Fig. 7 depicts higher fasting blood glucose levels and HOMA-IR index in germ-free WT mice receiving gut microbiota from HFD-fed NOD2-/- mice indicating exacerbated insulin resistance. Since this is a key finding of the present study and since the potential value of HOMA-IR to assess insulin resistance in mice is controversially discussed glucose as well as insulin tolerance tests should be performed in these mice.

Response: This is a tough, but fair question. The GTTs always prove difficult in these previously germ-free mice, where we consistently find that all mice are very glucose intolerant given only 4-5 weeks of HFD-feeding. We have actually repeated these experiments using an entirely new set of donors and germ-free recipients –and found the exact same result –all of the previously germ-free mice are very glucose intolerant –as such – there is little scope to find a difference. It is not clear how the GTT assessment is being affected in mice born and developing without bacteria (i.e. previously germ-free mice), but this is beyond the scope of the current study.

However, in both experiments HOMA-IR and fasting blood glucose is higher in WT, recipient, germ-free mice receiving the NOD2$^{-/-}$/HFD flora. It is true that HOMA-IR is not as informative in

term of mechanisms of glucose or insulin intolerance as dynamic tests (such as GTTs or ITTs)–but it is a valid measure in mice.

Importantly, we have now included new data using a dynamic measure of glucose control – a pyruvate tolerance test (PTT). The PTT shows a significant increase in blood glucose in WT, recipient, germ-free mice receiving the NOD2$^{-/-}$/HFD flora compared to those mice that received the WT flora. This supports our consistent finding throughout the paper of the involvement of the liver and coincides with increased markers of gluconeogenesis (G-6-P; Fig 7) in these NOD2-recipent mice. We have reinforced and stressed our comments and conclusions that the microbiota of NOD2$^{-/-}$ mice drive a subset of the metabolic phenotypes.

3) It is several times stated (including abstract, results and discussion sections) that NOD2 detection of PGN protects against HFD-induced insulin resistance, adipose tissue inflammation etc. However, in the present study, a loss-of-function rather than a gain-of-function model was used. Thus, the authors should claim that ablation of NOD2 detection of PGN aggravates HFD-induced insulin resistance, adipose tissue inflammation etc. and that NOD2 may play a protective role in the development of obesity-associated insulin resistance and inflammation. Furthermore, these results may suggest that forced NOD2 expression/NOD2 binding suppress the production of pro-inflammatory cytokines. However, previous studies including their own have suggested the opposite. What would be the authors' explanation for such apparent discrepancy.

Response: As suggested by the reviewer, we have modified the text in all of the relevant sections including the abstract, results and discussion to accurately describe the loss-of-function approach. Based on this comment we have also modified the title of the manuscript. The reviewer is correct that our data shows that a NOD2 deletion strategy exacerbated insulin resistance and inflammation and we have modified all of the relevant the text accordingly.

The reviewer has expertly pointed out "that forced NOD2 expression/NOD2 binding" may suppress inflammation, which appears to be conflicting with our previous paper and several others on the pro-inflammatory role of NOD2. We agree that activating NOD2 *in vivo* is a logical extension of this work (but beyond the scope of this manuscript). These experiments would have to directly test if/how activation of NOD2 could be insulin sensitizing through modulation of immunity and define the metabolic sites of action. There is precedence for NOD2 skewing immune responses toward Th2/M2 characteristics in the literature (J Clin Invest. 2008 Feb;118(2):545-59; Mucosal Immunol. 2014 Mar 26. doi: 10.1038/mi.2014.19.). Skewing immune responses to M2/Th2 characteristics is known to be associated with reduced insulin resistance –and we have started a comprehensive analysis of how NOD2 ligand engagement promotes insulin sensitivity. Indeed, we can show that ligand activation of NOD2 improves in vivo insulin sensitivity during obesity (unpublished, data not shown), but several mechanisms are involved and being investigated outside the scope of this manuscript.

The reviewer is correct that this finding is exactly opposite of what we originally hypothesized here and in our first paper showing that NOD2 activation promotes insulin resistance *in vitro* in clonal muscle cells (Tamrakar et al., Endocrinology, 2010). Based on all of our evidence *in vivo* and *in vitro*, we interpret that NOD2 action in clonal muscle cells does not adequately recapitulate the *in vivo* environment or that the contribution from direct sensing of NOD2 ligands in muscle cells is not important in the whole body response compared to effects in the gut, immune cells, liver and adipose tissue. We have discussed the implications of using a cell line versus whole body glucose control and the important difference in the "integrated role of inflammation, which in an appropriate situation, such as in acute phase, could prevent a low-grade chronic inflammatory state." (Burcelin, *Physiology* 27(5):300-7, 2012.)

4) Fig E4 depicts increased WAT mass in NOD2-/- mice compared to WT mice. Was such increase/difference observed for all depots e.g. perigonadal, inguinal, mesenteric, etc. or are NOD2 mice prone to mainly increase fat mass at visceral sites. Such analysis should be presented in Fig. 1.

Response: This is an important comment from the reviewer about increased fat mass in HFD-fed NOD2$^{-/-}$ mice compared to WT mice in Fig E4. We do not routinely measure every fat pad, but the increased mass of the paired gonadal fat pads shown in Fig E4 is mirrored by increased mesenteric fat mass in NOD2$^{-/-}$ mice compared to WT mice. The gut associated mesenteric adipose tissue seems very relevant to our study. According to the reviewer's comments, we have now included mesenteric adipose tissue (MAT) mass data in Figure E4.

It is very important that we weight-matched the mice in Figure 1 in order to demonstrate that NOD2-deletion exacerbates insulin resistance independently of any effect on obesity when compared to WT mice. We apologize for only showing the body mass data in Figure 1E. According to the reviewers comment, we have included the adipose tissue mass for paired gonadal and mesenteric depots in these weight-matched mice in Figure E1. We had to weight-match the mice in this case, since NOD2$^{-/-}$ mice actually eat less than WT mice (data not shown) and pair-feeding would not work in this model to study insulin resistance independently from changes in obesity.

Minor comments

1) 2nd sentence of 1st paragraph/introduction: increased neutrophil infiltration of adipose tissue in HFD-fed was first shown by Elgazar-Carmon V et al. (J Lipid Res 2008). This article needs to be cited here.

Thank you – this paper is now cited.

2) Fig. 1 E: Please provide time course for glucose infusion rate and glucose levels during the whole clamp period in a supplementary figure.

We have now included the time course data for GINF and blood glucose during the entire clamp protocol and indicated the "clamp period" used for calculations in Fig 1E. We have also reported the body mass for these weight matched mice that were clamped in Fig 1E.

3) Fig. 2F and 4I: Please show representative Western Blots for pAkt and pFOXO1.

We have now included this data according to multiple reviewer requests

4) Fig. 4E: The difference in liver lipids between the genotypes is convincing, especially when considering the TAG-data in Fig. 4F. However, one would expect that mice fed a HFD for 16 weeks would show some degree of hepatic steatosis, i.e. the presence of lipid droplets on H&E staining. Therefore the chosen microscopic image for control HFD-fed mice does not seem to be representative.

This is a good point. All of our HFD-fed mice displayed lipid droplets by histology, which is consistent with the TAG quantification in the livers. The main purpose of this image was to show macrophage content by IHC (i.e. F4/80^{+} staining) depicted by the red colour. We have now included a better, representative figure IHC figure in HFD-fed WT mice that also better depicts the relative changes in lipid droplet abundance and architecture between the genotypes after a HFD.

2nd Editorial Decision 09 January 2015

Thank you for the submission of your revised manuscript to EMBO Molecular Medicine. We have now received the enclosed reports from the referees that were asked to re-assess it. As you will see the reviewers are now globally supportive and I am pleased to inform you that we will be able to

accept your manuscript pending the following final amendments:

-please address the last comment of referee 2

Please submit your revised manuscript within two weeks. I look forward to seeing a revised form of your manuscript as soon as possible.

***** Reviewer's comments *****

Referee #2 (Remarks):

The authors adequately addressed my queries and I support the publication of the revised manuscript.

As a minor issue, there are two Figure 3D in the revised manuscript. Please correct the Figure label.

Referee #3 (Remarks):

Denou et al. did a good job to address most of my concerns and comments.

2nd Revision - authors' response 12 January 2015

Please address the last comment of referee 1 ... "As a minor issue, there are two Figure 3D in the revised manuscript. Please correct the Figure label."

We changed the figure lettering, which is now correct. Thank you.
